# Instrumentation and methods for efficient time-resolved X-ray crystallography of biomolecular systems with sub-10 ms time resolution

**DOI:** 10.1107/S205225252500288X

**Published:** 2025-04-25

**Authors:** John A. Indergaard, Kashfia Mahmood, Leo Gabriel, Gary Zhong, Adam Lastovka, Matthew J. McLeod, Robert E. Thorne

**Affiliations:** a Physics Department Cornell University 142 Sciences Drive Ithaca NY14850 USA; b Mechanical and Mechatronics Engineering University of Waterloo 200 University Avenue West Waterloo OntarioN2L 3G1 Canada; University of Virginia, USA

**Keywords:** time-resolved crystallography, enzyme mechanism, protein dynamics, mix and quench, time-resolved cryo-EM

## Abstract

Time-resolved X-ray crystallography has great promise to illuminate structure–function relations and key steps of enzymatic reactions with atomic resolution. The dominant methods for chemically-initiated reactions require complex instrumentation at the X-ray beamline, significant effort to operate and maintain this instrumentation, and enormous numbers (∼10^5^–10^9^) of crystals per time point. We describe instrumentation and methods that enable high-throughput time-resolved study of biomolecular systems using standard crystallography sample supports and mail-in X-ray data collection at standard high-throughput cryocrystallography synchrotron beamlines. The instrumentation allows rapid reaction initiation by mixing of crystals and substrate/ligand solution, rapid capture of structural states via thermal quenching with no pre-cooling perturbations, and yields time resolutions in the single-millisecond range, comparable to the best achieved by any non-photo-initiated method in both crystallography and cryo-electron microscopy. Our approach to reaction initiation has the advantages of simplicity, robustness, low cost, adaptability to diverse ligand solutions and small minimum volume requirements, making it well suited to routine laboratory use and to high-throughput screening. We report the detailed characterization of instrument performance, present structures of binding of *N*-acetylglucosamine to lysozyme at time points from 8 ms to 2 s determined using only one crystal per time point, and discuss additional improvements that will push time resolution toward 1 ms.

## Abstract 2

Methods and instrumentation for reaction initiation via mixing followed by rapid cooling allow sample-efficient time-resolved crystallographic studies with sub-10 ms time resolution. The instrumentation is robust, amenable to diverse samples, cost-effective and enables the remote collection of time-resolved X-ray data using standard sample supports and high-throughput cryocrystallography beamlines.

## 1. Introduction

Making ‘movies’ of molecules in action has been a longstanding goal in many fields of structural science. In structural biology, time-resolved (TR) studies of biomolecular response to light, ligand binding and changes in, for example, temperature or pH have been spurred on by developments in high-flux, high-brilliance X-ray sources – particularly X-ray free-electron lasers (XFELs) (Stagno *et al.*, 2017 ▸; Fuller *et al.*, 2017 ▸; Beyerlein *et al.*, 2017 ▸; Kupitz *et al.*, 2017 ▸; Olmos *et al.*, 2018 ▸; Mehrabi, Schulz, Agthe *et al.*, 2019 ▸; Pandey *et al.*, 2021 ▸; Butryn *et al.*, 2021 ▸) and high-frame-rate X-ray detectors for X-ray crystallography, and by dramatic improvements in detector technology, resolution and software for cryo-electron microscopy (Berriman & Unwin, 1994 ▸; Walker *et al.*, 1995 ▸; Chen *et al.*, 2015 ▸; Kontziampasis *et al.*, 2019 ▸; Klebl *et al.*, 2021 ▸; Amann *et al.*, 2023 ▸).

Time-resolved X-ray crystallography (TR-X; Moffat, 2001 ▸; Orville, 2018 ▸; Martin-Garcia, 2021 ▸; Brändén & Neutze, 2021 ▸; Hekstra, 2023 ▸; Schmidt, 2023 ▸) routinely yields near-atomic resolution, revealing changes in biomolecule and ligand structure (atomic positions) and dynamics (occupancies and *B* factors). Reaction initiation may be via direct optical excitation (Šrajer *et al.*, 1996 ▸; Schlichting *et al.*, 2000 ▸; Schotte *et al.*, 2003 ▸; Bourgeois & Weik, 2009 ▸; Maestre-Reyna *et al.*, 2023 ▸), applicable to the 1–2% of proteins with suitable activity, where time resolutions in the femtosecond range have been achieved (Barends *et al.*, 2015 ▸; Hosseinizadeh *et al.*, 2021 ▸). The vast majority of TR-X targets require chemical initiation by diffusing molecules (referred to here as ligands for simplicity) into the crystal or by optical uncaging of molecules previously loaded into the crystal (Tosha *et al.*, 2017 ▸; Mehrabi, Schulz, Dsouza *et al.*, 2019 ▸; Monteiro *et al.*, 2021 ▸; Wranik *et al.*, 2023 ▸).

For room-temperature data collection (Section S2), ligand solution may be dispensed as drops onto crystals (Mehrabi, Schulz, Agthe *et al.*, 2019 ▸; Mehrabi *et al.*, 2020 ▸) or may be mixed with crystal-containing streams using, for example, T-junction and coaxial flow microfluidic mixers (Stagno *et al.*, 2017 ▸; Dasgupta *et al.*, 2019 ▸; Calvey *et al.*, 2019 ▸; Pandey *et al.*, 2021 ▸). Crystals may then be translated using a motorized stage (Mehrabi, Schulz, Agthe *et al.*, 2019 ▸; Mehrabi *et al.*, 2020 ▸), a moving ‘tape’ (Beyerlein *et al.*, 2017 ▸; Butryn *et al.*, 2021 ▸), or a high-speed liquid jet (Pandey *et al.*, 2021 ▸) into the X-ray beam.

Temporal resolution Δ*t*_min_ is determined by the time interval Δ*t*_mix_ for mixing and ligand diffusion into the crystal, the time Δ*t*_trans_ for crystal translation into the beam, and the X-ray exposure time Δ*t*_exp_. Mixing and diffusion times will be crystal-, ligand- and temperature-dependent (Cvetkovic *et al.*, 2005 ▸; Geremia *et al.*, 2006 ▸; Schmidt, 2020 ▸) and may temporally overlap with crystal translation and X-ray exposure, so the actual temporal resolutions achieved are difficult to ascertain and the reported values are ‘nominal’ estimates based on simplifying assumptions – for example *in crystallo* diffusion coefficients equal to those in bulk solution (Pandey *et al.*, 2021 ▸; Cvetkovic *et al.*, 2005 ▸). Achieving high temporal resolution requires the use of small crystals (∼1–20 µm in minimum dimension).

In room-temperature TR-X using microcrystals, X-ray data are typically collected from each crystal in a single, unknown orientation. At XFELs, only one frame can be collected before a crystal and its surroundings vaporize. At synchrotrons, often only a few frames may be collected before room-temperature radiation-dose limits are exceeded. Crystal utilization can be extremely inefficient and complete data sets sufficient for structure determination may consume 10^5^–10^9^ crystals for each time point (Pandey *et al.*, 2021 ▸; Thorne, 2023 ▸). Special­ized hardware for mixing and sample delivery must be incorporated into the X-ray beamline, and data collection often requires multiple personnel on site. In part because of these complexities, the number of biomolecular systems that have been studied using TR-X has grown slowly; at the present rate (Brändén & Neutze, 2021 ▸), TR-X coverage of 1% of the proteins currently in the PDB will take a century.

An alternative approach to time-resolved structural studies involves initiating a reaction in the sample and then, after a time delay, rapidly cooling the sample to cryogenic temperature (Moffat & Henderson, 1995 ▸; Stoddard, 2001 ▸; Ding *et al.*, 2006 ▸; Clinger *et al.*, 2021 ▸). If the cooling time Δ*t*_cool_ [from the reaction temperature to below ∼200 K (the protein–solvent glass transition) or ∼150 K (the glass transition of the solvent)] is sufficiently short, many salient aspects of the reaction-temperature state may be captured. The cold sample may then be examined at another time and place. Unlike in room-temperature methods, the data-collection time is not constrained by, and can be much longer than, the reaction time point being probed. The temporal resolution Δ*t*_min_ is set by the mixing and diffusion time Δ*t*_mix_, the time for sample translation into the cooling medium Δ*t*_trans_, and the reaction quenching time Δ*t*_quench_ (<Δ*t*_cool_).

‘Mix-and-quench’ approaches have long been used in time-resolved spectroscopic and structural studies of biomolecular systems. In biomolecular cryo-EM (where the cryocooling of samples is essential), experiments in the 1990s yielded a nominal time resolution of ∼5 ms (Berriman & Unwin, 1994 ▸). Subsequent TR-cryoEM work has not improved upon this (Amann *et al.*, 2023 ▸; Section S3). In biomolecular crystallo­graphy (Section S1), experiments in the 1990s achieved time resolutions of tens of seconds to hours. In 2021, Clinger and coworkers demonstrated a mix-and-quench TR-X approach that yielded structures of the reaction of oxaloacetic acid (OAA) with the metabolic enzyme phosphoenolpyruvate carboxykinase (rat cytosolic PEPCK) with 40 ms nominal time resolution (Clinger *et al.*, 2021 ▸). An experiment in 2023 using a commercial drop-on-demand dispenser for reaction initiation yielded structures of binding of glucose and 2,3-butanediol to xylose isomerase with a less well defined time resolution of ~65 ms (Mehrabi *et al.*, 2023 ▸; Section S1).

Building on our 2021 demonstration experiment (Clinger *et al.*, 2021 ▸), we have designed and constructed a semi-automated sample-preparation system for mix-and-quench time-resolved crystallography that yields time resolutions comparable to the best achieved using any other chemically triggered TR-X or TR-cryoEM approach. This system uses crystals held on standard crystallography supports in standard crystallo­graphy bases, allows the deposition of ligand-containing solution with high reliability and time precision, quenches reactions in a few milliseconds, facilitates manual post-cooling storage of samples in standard crystallography pucks, and minimizes the consumption of both ligand and protein. We demonstrate the application of this instrument and method to the binding of *N*-acetylglucosamine (NAG1) and lysozyme. Structures showing the evolution of ligand binding were obtained at seven time points from 8 ms to 2 s using only a single crystal per time point and 6 h of remote cryocrystallographic data collection. With further optimization, the nominal time resolution can be reduced to ∼2 ms (Section 5.2). Our reaction-initiation mechanism is simple, inexpensive and robust and can be implemented on both automated and manual plunge coolers. Our approach is particularly well suited to routine home laboratory use and to sample-efficient high-throughput screening of targets for subsequent room/biological temperature TR-X study.

## 2. Results

### 2.1. System design parameters and constraints

The design objectives for our system are described in the supporting information (Section S4). Key objectives included (i) a cooling time from 273 to 200 K of <5 ms for sub-30 µm samples, (ii) a minimum time between ligand-solution deposition and sample cooling in boiling liquid nitrogen (LN_2_) of <10 ms, (iii) a sample temperature held to within ±5 K of its initial value to within 2 mm of the LN_2_ surface, (iv) a ligand-solution temperature held to within ±5 K of the initial sample temperature, to ensure that ligand binding and biomolecule response occur at constant temperature prior to quenching, (v) complete compatibility with all sample supports, tools and hardware for high-throughput cryocrystallography, and (vi) the execution of all steps required for sample preparation by a single person.

### 2.2. System components

**Figure 1:**
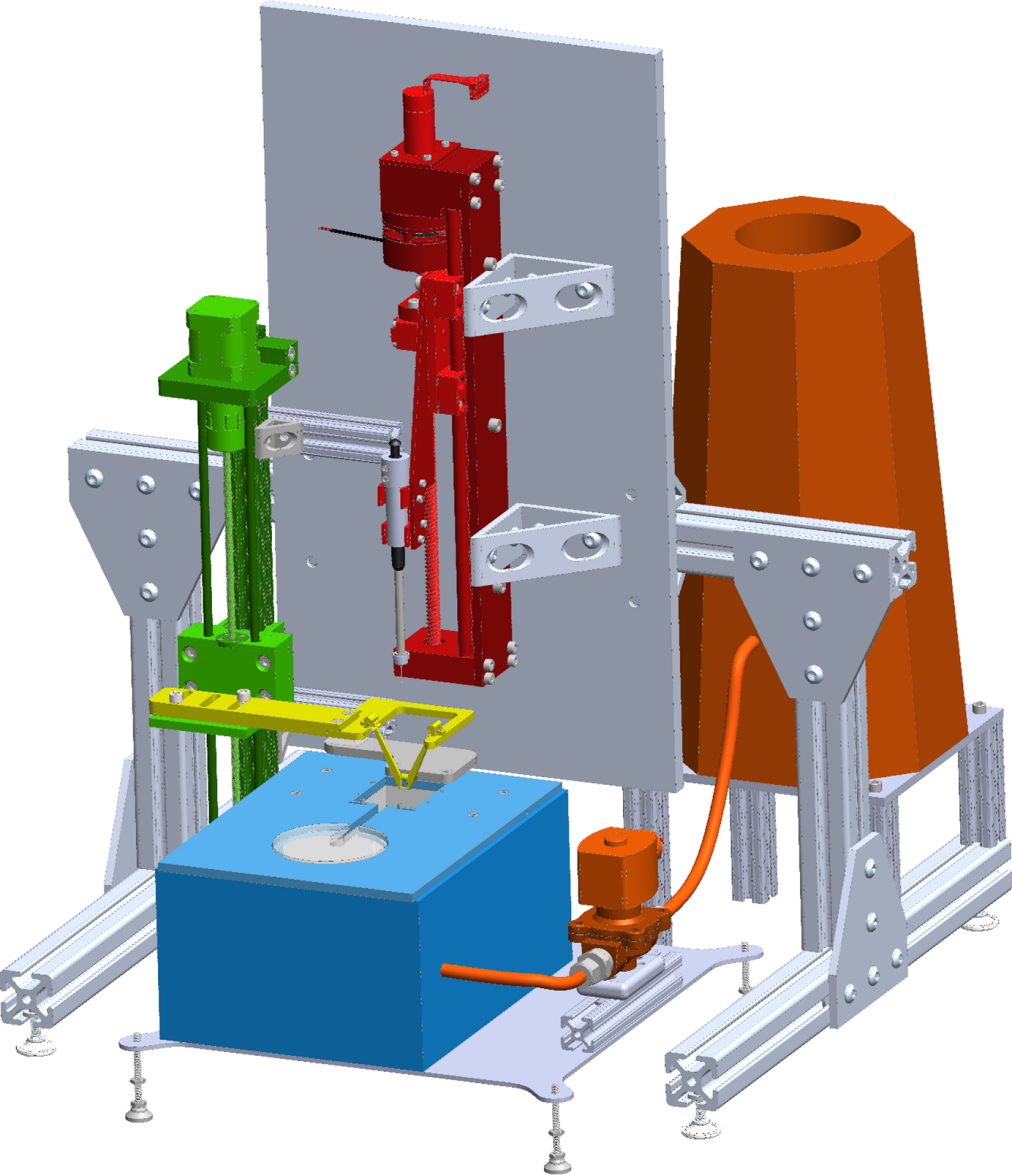
Schematic illustration of the mix-and-quench sample-preparation system for time-resolved X-ray crystallography (TR-X). Electronics, electrical and gas connections are not shown. Red, sample vertical translation stage; green, deposition vertical translation stage; yellow, sample-deposition platform; blue, sample cryocooling reservoir with gas-exchange manifold; orange, liquid-nitrogen storage and fill system. Supplementary Fig. S1 shows a photograph of a recent version of the system.

Fig. 1 ▸ shows the overall design of an automated mix-and-quench sample-preparation system that meets our design objectives, and Supplementary Fig. S1 shows photographs of an implementation of the system. Major system components include a high-speed vertical translation stage for sample plunging, a sample-holding wand and carriage that attach to the vertical stage, an LN_2_-filled sample-cryocooling and storage reservoir holding a cryocrystallography sample storage ‘UniPuck’, a gas-exchange manifold (GEM) defining a sample plunge bore that controls the gas present above the LN_2_ surface within the bore, a LN_2_-level control system, a ligand-solution transfer/reaction-initiation mechanism, a low-speed vertical translation stage holding the ligand solution-transfer mechanism, and an interface unit and computer for system control.

#### 2.2.1. Sample vertical translation stage

The high-speed vertical translation stage (Supplementary Fig. S2) was adapted from a design provided by MiTeGen (see conflict of interest statement) and uses a servo-motor-driven lead screw to accelerate a sample carriage to speeds of up to 3 m s^−1^. The sample carriage is guided by a double rail, reducing side-to-side motion/wobble as the lead screw rotates. Readouts from the motor encoder are processed by the motor controller to generate real-time data on sample-carriage height and speed. The motion can be stopped once the sample has entered the LN_2_ via both programmed motor deceleration and by an electromagnetically actuated brake.

A standard magnetic wand for cryocrystallography with push-button release [Supplementary Fig. S3(*a*)] holds the magnetic steel goniometer base [Supplementary Fig. S3(*d*)] into which the sample support is inserted [Supplementary Fig. S3(*e*)]. The wand is held in an adapter [Supplementary Figs. S3(*b*) and S3(*c*)] that is mechanically and magnetically captured by the plunge stage’s sample carriage.

#### 2.2.2. Sample cryocooling and storage reservoir

Supplementary Fig. S4 shows the design of the sample-cooling and storage reservoir. The reservoir base [Supplementary Fig. S4(*b*)] is machined from high-density polyurethane foam. It includes a circular recess with an orientation pin for the placement of a UniPuck (which holds up to 16 samples) and a through-hole in its sidewall for an LN_2_ fill tube. Raised cuboids at one end define a channel into which the sample is plunged and through which it is translated before insertion into the sample puck. The cuboids reduce the LN_2_ volume required to fill the reservoir to about 1 litre.

To prevent moisture condensation and frost accumulation, the reservoir has a heated lid [Supplementary Fig. S4(*c*)]. PID-controlled heaters embedded within the body of the lid maintain its surface at ∼298 K. The lid has an aperture for the plunge bore that connects via a slot to a larger aperture through which the cryocrystallography puck is loaded and removed. The puck aperture is covered by a clear, slotted, freely rotatable acrylic window. After each sample has been plunged through the plunge bore, the sample wand is removed from the plunge stage’s sample carriage, translated into the slotted portion of the acrylic window and rotated (with the window) into position above an open puck receptacle. The sample is then released from the wand and into the puck. This configuration allows all post-plunge sample motions to be completed with the sample remaining in LN_2_, minimizing the risk of accidental sample warming.

#### 2.2.3. Gas-exchange manifold

The gas-exchange manifold (GEM) serves multiple purposes. Firstly, it defines the plunge bore and isolates a small portion of the LN_2_ surface within the bore from the surrounding liquid surface, reducing waves to maintain a flat interface. Secondly, it removes cold gas present within the bore that naturally forms above the LN_2_ surface and replaces it with ambient-temperature N_2_ gas, minimizing precooling of the sample in cold gas before it enters the LN_2_ (Warkentin *et al.*, 2006 ▸). Thirdly, it minimizes the infiltration of warm moist room air into the sample-cryocooling reservoir and reduces frosting on all cold surfaces. The design of the manifold should allow placement of the liquid film used for reaction initiation as close to the LN_2_ surface as possible, as this determines the minimum time point after reaction initiation that can be probed.

Supplementary Fig. S5 shows one implementation of the GEM, which attaches to the underside of the lid above the cuboids. Two opposing rectangular cross-section channels slope downwards to where they intersect the plunge bore. These are supplied through electronically controlled valves by vacuum (provided by a compressed-gas vacuum generator) and dry room-temperature N_2_ gas (from an N_2_ cylinder) via connections in the rear of the GEM. For efficient cold gas removal and replacement, the LN_2_ fill level in the plunge chamber must lie within the vacuum and N_2_ gas channels where they meet the bore. A metal block containing a heater and temperature sensor inserts into the top of the GEM. It defines the top surfaces of the vacuum and make-up gas channels and maintains a constant bore wall temperature.

During operation, valves on the vacuum and make-up gas lines automatically open a pre-programmed time interval (∼0.5 s) before a plunge, removing cold gas, eliminating sample precooling and ensuring that the reaction proceeds at ambient temperature until the sample enters the LN_2_. For early reaction time points which require reaction initiation close to the LN_2_ surface, the GEM minimizes conductive and convective cooling of the ligand solution.

The GEM has a through-slot connecting the bore to the portion of the sample-cooling and storage reservoir holding the UniPuck, with a swing door sealing off the bore prior to and during plunges. This allows the sample wand to be translated through LN_2_ from the bore and into the puck.

#### 2.2.4. LN_2_ level control

An LN_2_ storage reservoir (Fig. 1 ▸) connects through an insulated pipe to an electrically-actuated valve that feeds LN_2_ into the plunge reservoir. The LN_2_ pipe enters near the bottom of the chamber to minimize surface wave production. Filling is controlled through the GUI and allows the LN_2_ level to be maintained within ±2 mm, which is adequate to ensure efficient cold gas removal and replacement and accurate time-point determination.

#### 2.2.5. Ligand-solution deposition stage

Hardware for depositing ligand solution onto samples (plunge-through-film or drop-on-demand) is attached to a second, stepper-motor-driven vertical translation stage: the ligand-solution deposition stage (Supplementary Fig. S6). The stage allows the height *h* of liquid deposition above the LN_2_ surface and thus the time between liquid transfer/reaction initiation and contact with LN_2_ to be varied under computer control.

### 2.3. Ligand-solution deposition system

A large portion of our development work has focused on the design and optimization of methods for depositing ligand-containing solution onto samples held on thin-film crystallo­graphy supports or on automounter-compatible serial crystallography supports.

Initial experiments in 2020 reported by Clinger *et al.* (2021 ▸) used a ‘drop dispense and plunge’ approach similar to that used in most TR-cryoEM experiments (Section S3) and to that used by Mehrabi *et al.* (2023 ▸) for TR-X (Section S1). Samples on microfabricated supports were loaded within a humidified ‘tent’ into a NANUQ automated plunge cooler (MiTeGen). A commercial drop-on-demand dispenser (Microdrop Technologies MD-K-130) dispensed 150 pl drops onto the sample support and the sample was plunged after a variable delay into LN_2_. To ensure that randomly located crystals on the sample support were covered, dispensed volumes of at least several nanolitres and dispensing of at least ∼50 drops were required. At the maximum dispense rate of ∼2 kHz, the dispensing time was at least 25 ms. Acceleration of the sample from rest to ∼2 m s^−1^ upon entering the LN_2_ (to ensure fast cooling) required ∼100 ms. Consequently, the nominal time resolution was ∼112 ± 12 ms (average and spread over the sample support), larger than the turnover rates of many enzymes and inadequate to see intermediate states along their reaction/response pathway. In Mehrabi *et al.* (2023 ▸), dispensing of 200–250 drops with volumes of 150 pl at 5 kHz took 40–50 ms, crystal plunging after dispensing took ∼40 ms and the nominal time between reaction initiation and cooling was ∼65 ± 25 ms.

An alternative dispensed-drop approach that can give much better time resolution is to dispense a single large (∼2 nl) drop at high speed towards a sample support plunging at high speed towards the LN_2_. For 10 ms time resolution, this requires hitting an ∼200 µm target moving downward at 2 m s^−1^ with a ∼150 µm drop moving horizontally at a few m s^−1^ within 2 cm of the LN_2_ surface. This is technically feasible and currently under development in our laboratory. Commercial drop dispensers meeting the required specifications cost at least 20 000 USD, other required system components add to this cost, and dispensing parameters must be optimized for each solution to ensure reproducibility.

We have thus pursued the simpler and less costly plunge-through-film approach that we first reported in 2021 (Clinger *et al.*, 2021 ▸). There, samples were plunged through a film of ligand solution spanning a 10 mm diameter wire loop positioned on the plunge path at height *h* above the LN_2_ surface, yielding a nominal time resolution of 40 ms. Ligand-solution films in loops of that diameter were unstable unless a small amount of detergent (sodium dodecyl sulfate, SDS) was added. Solution transfer to sample supports was unreliable, with less than one in four plunged sample supports having a suitable amount and distribution of solution. As standard sample-holding goniometer bases did not fit through the wire loop, sample supports were held by a 3 mm diameter post that was inserted after plunging into a standard diameter base. Manual assembly of the two parts in LN_2_ proved cumbersome and the magnet holding the post to the base led to faults during beamline automounting.

We have designed and tested several alternative plunge-through-film approaches for use with standard crystallography sample supports and goniometer bases and that can be reliably used in TR-X sample-preparation systems of varying complexity and cost.

Supplementary Fig. S8 shows the plunge-through-film configuration used to prepare most samples for the TR-X experiments discussed in Section 4. A 3 mm inner diameter (ID) film-holding loop was formed by two mating halves projecting from ‘saloon doors’. The doors were held in the closed, loop-forming configuration using small embedded magnets. After the sample support passed through the film, impact of the goniometer base with the doors pushed them downwards and out of the way, allowing the base and sample wand to continue their plunge unimpeded (Supplementary Movie S1). The 3D-printed doors were lightweight and strong, surviving hundreds of impacts without breakage or noticeable degradation. The platform holding the doors could be lowered into contact with the top of the gas-exchange manifold, the ∼24 mm height of which above the LN_2_ surface was set by the minimum height required for the doors to swing open. This placed the loop ∼50 mm above the LN_2_ surface, giving a minimum time point of about 20 ms. For the 8 ms time-point data in Section 4, a break-away loop that dropped into the plunge bore of the GEM (Supplementary Fig. S7) was used.

**Figure 2:**
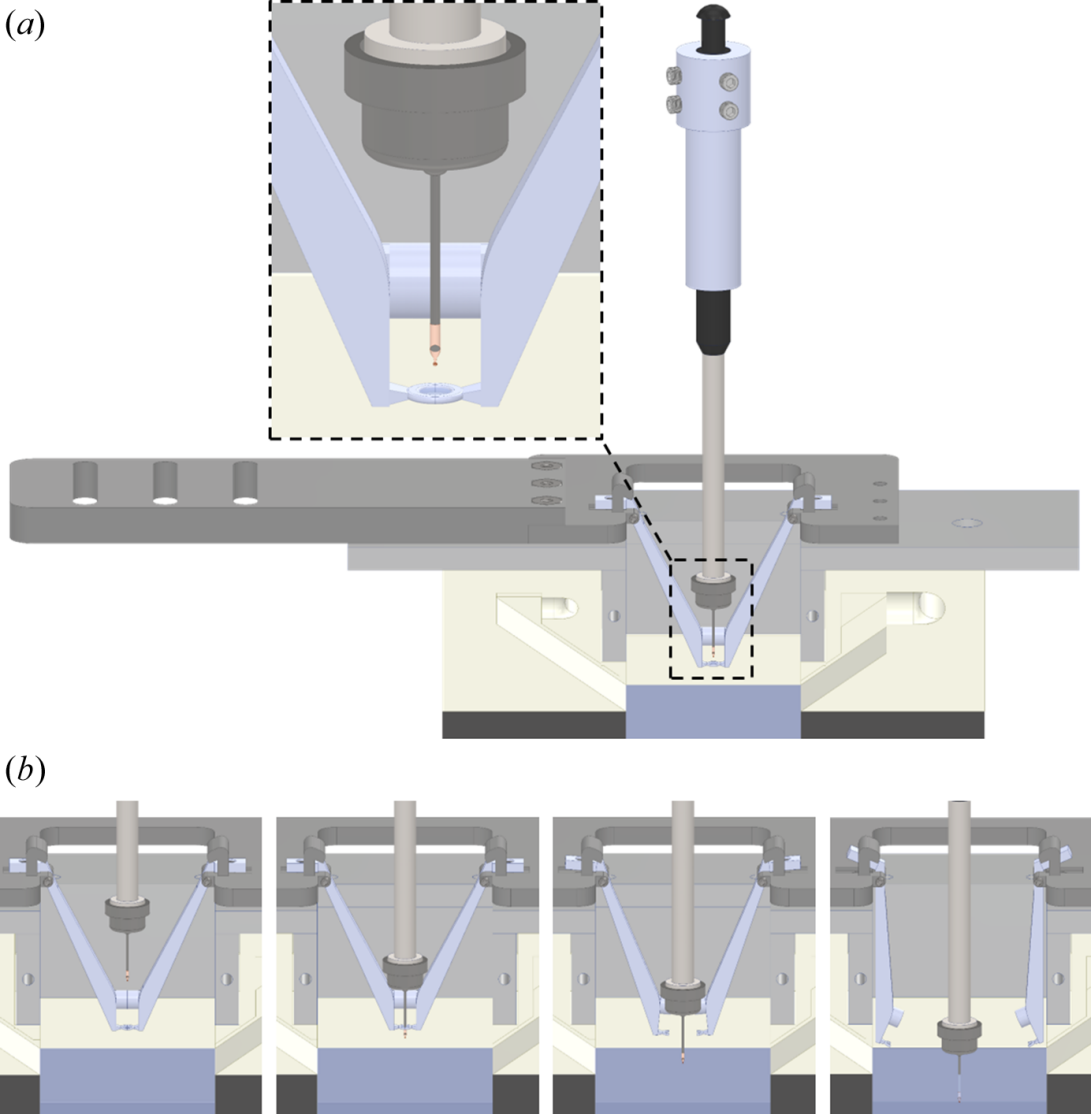
Plunge-through-film ligand-solution deposition mechanism suitable for achieving few-millisecond time points. (*a*) Roughly 2 µl of ligand solution is loaded into a 2 mm diameter loop. The loop is formed from two interlocking halves attached to hinged arms, which are held together by opposing magnets in each arm. The arms project downwards from the platform that attaches to the deposition vertical translation stage (Fig. 1 ▸ and Supplementary Fig. S6) and fit within the plunge bore. When the platform is moved to its lowest position, the loop is positioned ∼2 mm above the LN_2_ surface, corresponding to a nominal time point of 1 ms. (*b*) Liquid is transferred onto the samples and support as they pass through the ligand-solution film spanning the loop. Glancing impact of the gonio­meter base with the hinged arms forces them apart. With the arms out of the way, the sample, base and wand continue to the LN_2_.

Fig. 2 ▸ shows our current plunge-through-film configuration, which allows the time interval between ligand-solution deposition and sample entry into LN_2_ to be as small as 1 ms. The 2 mm ID loop is formed by two mating halves attached to hinged arms that project downwards from the platform and into the plunge bore of the GEM. With our current GEM design, this allows the loop to be brought within ∼2 mm of the LN_2_ surface. The arms are held closed using magnets, and their motion when opening is largely horizontal, keeping the loop out of the LN_2_. The angled arms are gradually rather than abruptly pushed apart by glancing contact with the downward-travelling goniometer base, reducing peak forces (Sup­plementary Movie S2).

### 2.4. System control and software interface

Nearly all system functions are controlled using custom scripts in Python running on a desktop computer and communicating with instrument components through a National Instruments data-acquisition (NI-DAQ) system consisting of a terminal block (NI SCB-68A) for distributing signal inputs/outputs and a multifunction I/O board (NI PCIe-6361) for signal processing.

Supplementary Figs. S9 and S10 show screen shots of the graphical user interface (GUI) used to control the system. The GUI has six tabs, which are described in detail in Section S5. The ‘Manual Plunge’ tab enables manual control of the sample vertical translation stage and the ligand-solution deposition stage as well as all peripheral devices, including lid and gas-exchange manifold heaters, vacuum and dry N_2_ make-up gas valves, the cryogenic LN_2_ fill valve, and the sample-stage brake. It also allows logging of plunge-velocity and temperature profiles (versus time and height *h*), with the latter recorded using a thermocouple incorporated into a calibration base that mounts on the sample wand.

The ‘Auto Plunge’ tab facilitates sample preparation by automating the setup to achieve a desired time point. The user selects a predefined motion profile, a desired time point and options such as homing after plunging. On pressing ‘SETUP’, the software determines whether a simple plunge or (for long time points) a step–pause–plunge motion is required and positions the deposition stage at the required height. Pressing ‘Plunge’ then executes the required motions and valve openings/closings.

The ‘Visualization’ tab allows the position, velocity and temperature data recorded during plunges to be plotted, with the LN_2_ level and deposition-stage heights indicated on the plots.

The ‘Utility’ tab allows several predefined routines that characterize system performance [temperature profiles, timing reproducibility, timing delays, and plunge stage motion reproducibility (*e.g.*Supplementary Fig. S12)] to be run automatically.

Heaters in the GEM and its lid are controlled by two PID controllers connected to power supplies mounted on the front of the instrument so that their temperature displays and buttons are readily accessible. Heater controller settings rarely need adjustment, so such adjustments are peformed manually.

### 2.5. System operation

During system initialization, the heaters are powered on to bring the GEM bore and lid to 25°C and prevent frosting when filling the sample plunge reservoir with LN_2_. A sample-storage puck (UniPuck) is placed in the plunge reservoir and locked in the correct orientation. The LN_2_ storage reservoir is filled, with the valve connecting it to the plunge reservoir closed. LN_2_ is directly added to the plunge reservoir until the sample-storage puck has fully cooled. The valve to the storage reservoir is opened and additional LN_2_ is added to the plunge reservoir until a target level within the GEM bore is reached.

Using the GUI, settings for sample preparation (plunge velocity profile, plunge type, time delay, *etc.*) are selected. The plunge stage is homed and the deposition stage is moved to an initial height for ligand-solution loading.

Crystals are harvested onto a microfabricated thin-film sample support (for example, a MicroCrystal Mount from MiTeGen) held in a standard goniometer base, and excess mother liquor is wicked or blotted away so that deposited ligand solution will immediately contact the crystals, minimizing the mixing/diffusion time. Ideally, harvesting and blotting should be performed in an environment humidified to at least 80% relative humidity (r.h.) or in a cold room. The sample support may then be placed in a cryovial with a wet sponge to maintain hydration during the time interval (<30 s) between harvesting and plunging. Roughly 1–2 µl of ligand solution cryoprotected with, for example, 5%(*w*/*v*) PEG 4000 and 10%(*v*/*v*) PEG 400 is deposited in the loop using a pipette and the cryovial (if present) is removed from the sample.

The fully automated plunge sequence is then initiated via the GUI. The deposition stage moves the loop to its target height *h*, vacuum and room-temperature N_2_-supply valves are opened to replace cold gas present within the plunge bore with room-temperature N_2_ gas and remain open until the sample is submerged in LN_2_, and immediately thereafter the sample is plunged through the ligand-solution film and loop and into LN_2_. The sample-holding wand is manually detached from the plunge stage, moved through the GEM channel and into the slot in the lid’s transparent window, rotated and translated into place above a receptacle in the UniPuck, and the sample is deposited. The wand may be warmed and dried using a warming station (Supplementary Fig. S11) before loading the next sample.

When plunging from the home position, the available vertical travel of the sample-deposition stage to the LN_2_ together with the default plunge stage velocity–time profile (chosen to minimize *t*_cool_) give a maximum time point of roughly 150 ms. For longer time points, the control software automatically positions the deposition stage so that the loop is 2.5 mm below the ‘home’ position of the sample, steps the sample about 5 mm through the loop and ligand-solution film in ∼30 ms (Supplementary Fig. S14), pauses an appropriate time and then plunges the sample the remaining distance into the LN_2_, minimizing *t*_cool_. Longer time points can also be accessed by plunging (without pausing) at a smaller speed *v*, but this will increase *t*_cool_ by a factor roughly proportional to *v*^1/2^.

To capture the earliest (few milliseconds) time points, the loop must be placed within millimetres of the LN_2_ surface. At this distance, the ligand solution will cool and eventually freeze: quickly if cold gas is allowed to accumulate in the plunge bore and more slowly (via radiation) if the cold gas is removed. To prevent this, during ligand-solution loading the loop is initially positioned ∼4 cm above the LN_2_ surface, above the GEM. During the plunge sequence, the stage translates the loop in <1 s to its target height *h* immediately before sample plunging.

All relevant information about each sample plunge, including position versus time, set time point and actual time point determined from the motion profile and deposition stage position, is automatically recorded. If the user decides to save this information and store the plunged sample in the puck, the GUI requests input of a sample name and other metadata and indicates the puck position to which the sample should be transferred.

## 3. System characterization and performance

### 3.1. Plunge velocities and profiles

**Figure 3:**
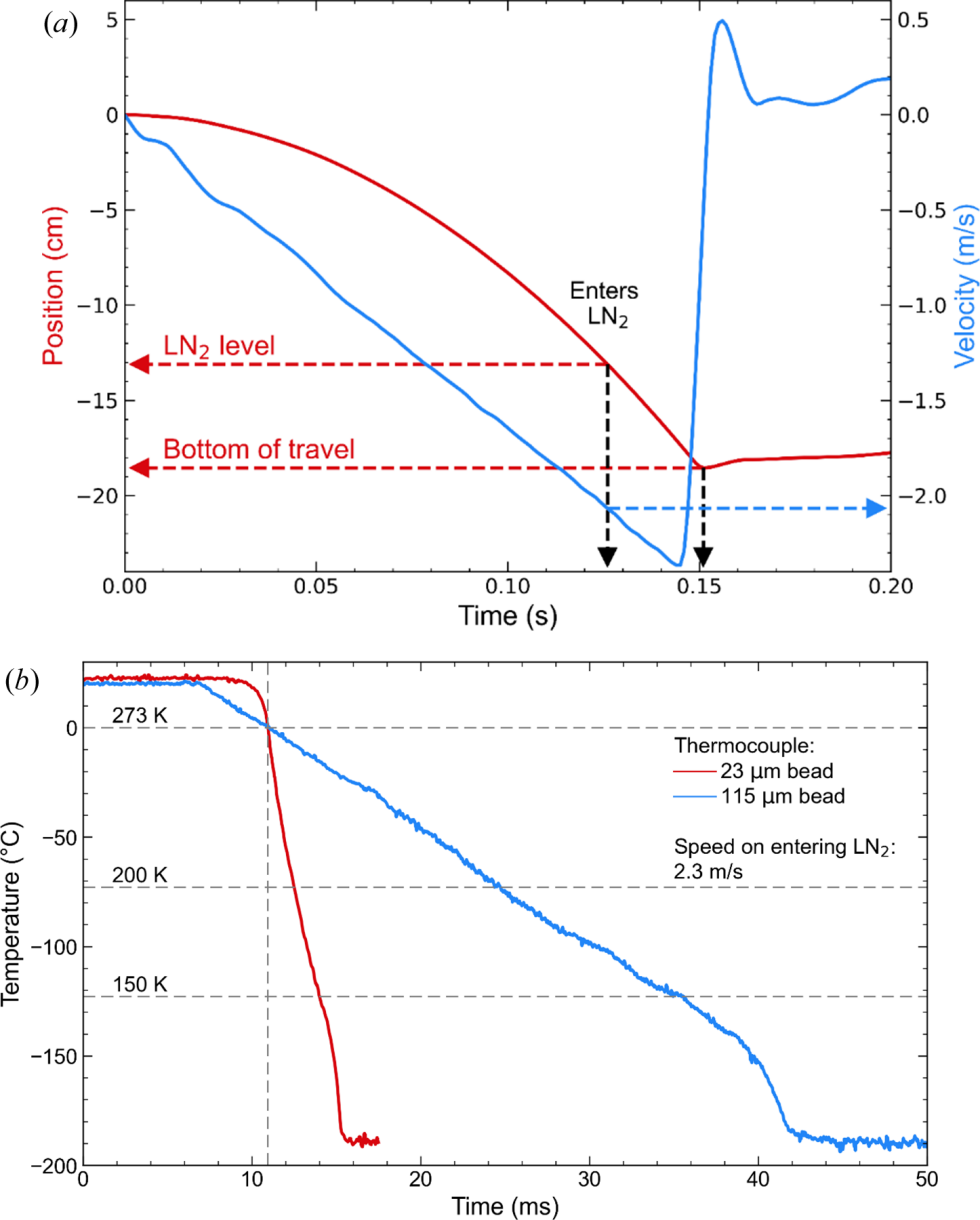
(*a*) Sample position and velocity versus time during a plunge into LN_2_, as recorded using the motor encoder of the sample vertical translation stage. The position of the LN_2_–gas interface and the hard stop of the vertical stage are indicated by horizontal dashed lines. In this case, the speed on entering the LN_2_ is 2.3 m s^−1^ and the brake was not applied. (*b*) Temperature versus time during a plunge as in (*a*) recorded using a thermocouple with a 23 µm bead and 12.5 µm leads and using a thermocouple with a 115 µm bead and 75 µm leads, with a speed on entering the LN_2_ of 2.3 m s^−1^. The corresponding cooling rates between 273 and 200 K are 46 ×10^3^ and 5.3 × 10^3^ K s^−1^, respectively, and those between 273 and 150 K are 40 × 10^3^ and 5.0 × 10^3^ K s^−1^, respectively. Assuming a power-law scaling of cooling rate with bead size, the corresponding cooling times for a 60 µm thick sample will be roughly 4.2 ms to 200 K and 8.0 ms to 150 K.

Fig. 3 ▸(*a*) shows the sample position and speed versus time for a full plunge with a speed on entering the LN_2_ of 2.0 m s^−1^. Supplementary Fig. S12 shows the recorded sample position and computed speed and acceleration versus time when the plunge stage was programmed to deliver a nominal reaction time (the travel time between the ligand film and the LN_2_) of 25 ms. Data were recorded for ten successive plunges, and the solid dark lines and shading above and below them indicate the average and full variation over the ten plunges for each quantity. The motion is extremely reproducible, with the measured nominal reaction time having an average of 26.8 ms with less than ±1 ms total variation. Variations of the LN_2_ level within the plunge dewar of ±2 mm give an additional uncertainty of roughly ±1 ms. Supplementary Fig. S13 shows the nominal reaction time (the transit time between the ligand-solution film and the LN_2_ surface) versus the loop height above the LN_2_ surface corresponding to the position–time profile in Supplementary Fig. S12. Supplementary Fig. S14 shows the sample position and velocity versus time during a 0.5 cm ‘step’ used in translating the sample through the loop and liquid film before pausing and then plunging in a step–pause–plunge sequence used to obtain long time points.

Side-to-side ‘wobble’ of the plunge stage during its motion from its home position to the LN_2_ surface was measured to be ∼0.7 mm when the sample was plunged at 1.8 m s^−1^ without braking and ∼1.4 mm when plunged with full braking (Section S6). The wobble trajectory was repeatable from plunge to plunge so that a sample support could be reliably directed through a 2 mm diameter loop without hitting it. Wobble primarily arose from warp in the lead screw as manufactured, which can be corrected.

### 3.2. Temperature profiles, cooling rates and LN_2_consumption

Fig. 3 ▸(*b*) shows temperature versus time measured during plunging of ∼23 µm (KFG-13, ANBE SMT) and ∼115 µm (CHAL-003, DwyerOmega) bead thermocouples into LN_2_ using the position/velocity profile shown in Fig. 3 ▸(*a*). The cooling times from 273 K to ∼200 K (near the protein–solvent glass transition where enzymatic activity ceases; Ringe & Petsko, 2003 ▸; Frauenfelder *et al.*, 2009 ▸; Doster, 2010 ▸) are 1.5 and 13 ms, respectively, corresponding to average cooling rates of ∼47 000 and 5600 K s^−1^, respectively. Scaling these results (Kriminski *et al.*, 2003 ▸) to an ∼50 µm thickness of deposited ligand solution typical of samples used for structure determination in Section 4, the corresponding cooling time is ∼4.2 ms and the time to quench the reaction is ∼2 ms (Section S9). Measurements using the smaller bead thermocouple show that, because of the action of the GEM, the gas temperature within the plunge bore remains within 10 K of its value at the initial position of the sample to within 0.4 mm of the LN_2_ surface.

The LN_2_ level within the plunge reservoir drops roughly 1 mm every 6 min and so requires topping up via the GUI roughly once every 10 min to maintain an appropriate level within the GEM. The sample-cryocooling reservoir is well isolated from ambient air, so that the system can be operated for ∼2 h without noticeable frost/ice accumulation. The contribution to accumulated frost of ligand solution projected downwards from the loop and into the plunge bore during each plunge is negligible.

### 3.3. Ligand-solution deposition: optimization and performance

Initial plunge-through-film experiments yielded irreproducible transfer of ligand-containing solution to the sample supports. Extensive experimentation using high-speed imaging during plunging to observe the liquid-transfer process and post-quench imaging to assess final ligand-solution distributions explored factors including ligand-solution properties, sample-support shape/design and hydrophobicity/hydrophilicity, and loop size and shape (Section S7).

Ligand solutions must contain cryoprotective agents (CPAs) to inhibit crystallization during plunge-cooling. These typically increase viscosity and decrease surface tension and so tend to increase liquid transfer. We obtained good performance using 5%(*w*/*v*) PEG 4000 and 10%(*v*/*v*) PEG 400. Robust liquid transfer was achieved using MicroCrystal Mount model #1 (MiTeGen), and this support design was used in the TR-X experiments described in Section 4. The polyimide support film has a ∼5 µm thick, 200 µm diameter active area with an array of 10 µm holes that allow back-side blotting. The rest of the support film, including a 40 µm wide ‘rim’ surrounding the active area, is ∼10 µm thick. When liquid is transferred to the support film, the step edge between the active area and the ‘rim’ acts to pin a receding liquid contact line (Kalinin *et al.*, 2009 ▸) and so keep the active area covered. Supplementary Fig. S15 shows example images of samples prepared in this way using 3 mm ID loops loaded with roughly 7.5 µl ligand solution. Analysis of these images (Section S8) indicates a typical transferred volume of ∼2 nl. Examples of liquid dynamics as a sample and support plunge through ligand-solution films spanning loops as in Fig. 2 ▸ and Supplementary Fig. S8 are shown in Supplementary Movies S1 and S2.

Ligand-solution film thicknesses were estimated to be <500 µm. The speed of the sample support as it passes through the ligand-solution film depends on the time point examined and varies between ∼2 m s^−1^ for shorter time points and ∼0.2 m s^−1^ (during a ‘step’ in a step–pause–plunge sequence) for long time points, so that the time taken to traverse 500 µm is ∼0.25 and 2.5 ms. Despite this wide variation in speed, the amount and pattern of liquid transfer to the sample support was similar. This suggests that the relevant dynamics may be dominated by wetting of the ligand-solution film to the polymer sample-support film.

## 4. System application: time-resolved crystallography of binding of NAG1 (GlcNAc) in lysozyme crystals

We prepared samples to observe the time-dependent binding of NAG1 (GlcNAc) in crystals of tetragonal hen egg-white lysozyme (HEWL), a model system used in evaluating TR-X methods (Mehrabi, Schulz, Agthe *et al.*, 2019 ▸; Beyerlein *et al.*, 2017 ▸; Butryn *et al.*, 2021 ▸). Samples containing crystals with minimum dimensions of 10–15 µm were prepared at eight time points between *t* = 0 and 2 s, with a minimum time point of 8 ms. These nominal time points are equal to the time between sample passage through the ligand film (which is complete in <1 ms) and entry into LN_2_ (Section S9). Samples were shipped to NSLS-II for remote data collection on the FMX beamline. Details of sample preparation and of data collection, analysis and modelling are described in Section S10.

**Figure 4:**
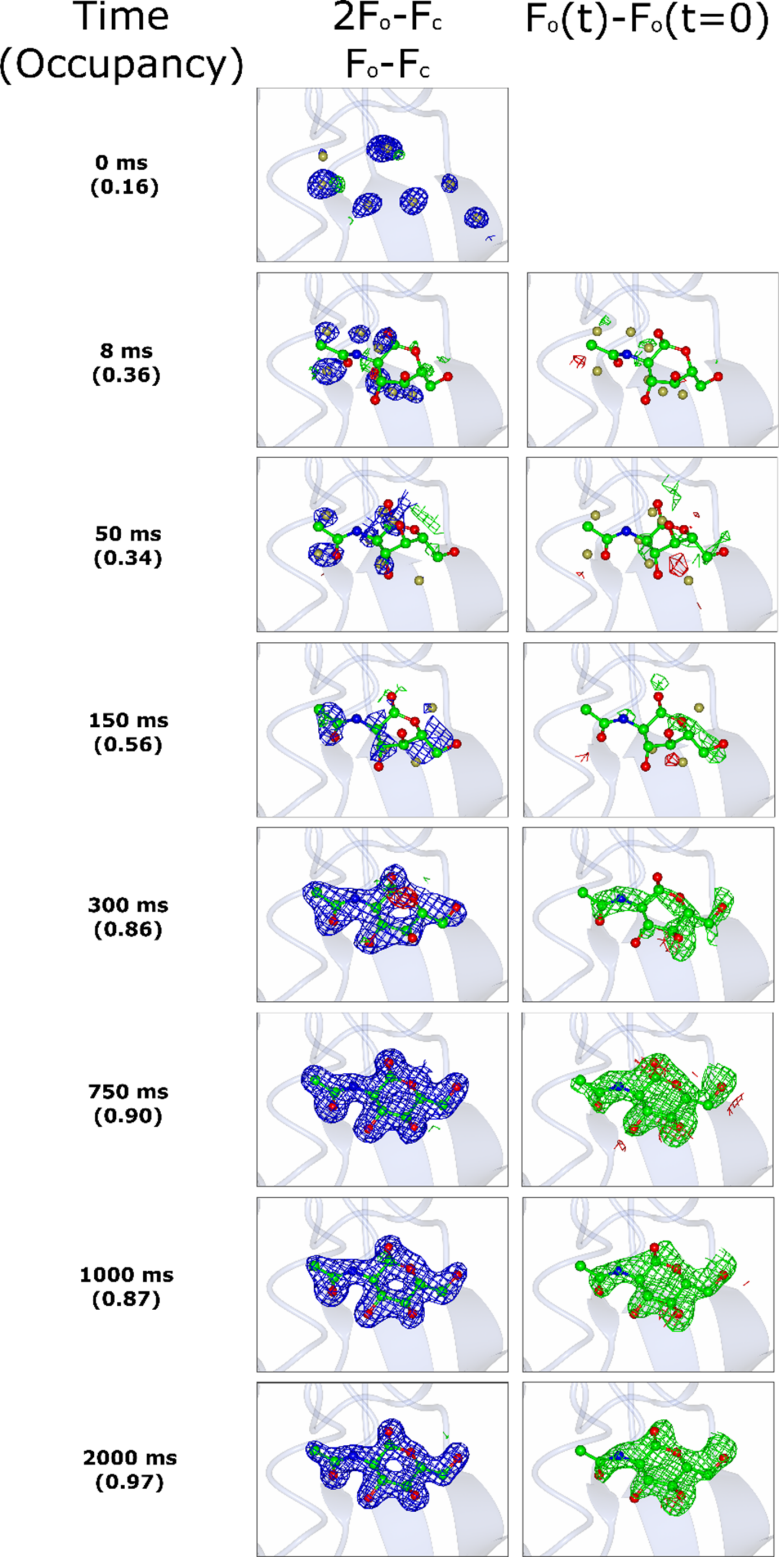
Time-resolved binding of NAG1 to hen egg-white lysozyme. Panels show electron density (2*F*_o_ − *F*_c_, contoured at 1σ), difference density (*F*_o_ − *F*_c_, contoured at 3σ) and isomorphous difference density (*F*_o_ − *F*_o_, contoured at 2.5σ) of NAG1 binding. Refined occupancies are indicated in parentheses. Although 0, 8 and 50 ms refinements give nonzero occupancy, there is no evidence for binding, even at lower σ electron-density thresholds.

Fig. 4 ▸ shows electron-density maps of the binding of NAG1 to HEWL and Supplementary Tables S1 and S2 summarize details of data collected at each time point. Although there is nonzero difference density and finite calculated occupancy at *t* = 0 s, 8 and 50 ms, there is no clear evidence of binding in either 2*F*_o_ − *F*_c_ or *F*_o_ − *F*_o_ maps at these times. At longer times binding is clearly observed and occupancy steadily increases towards 1.

**Figure 5:**
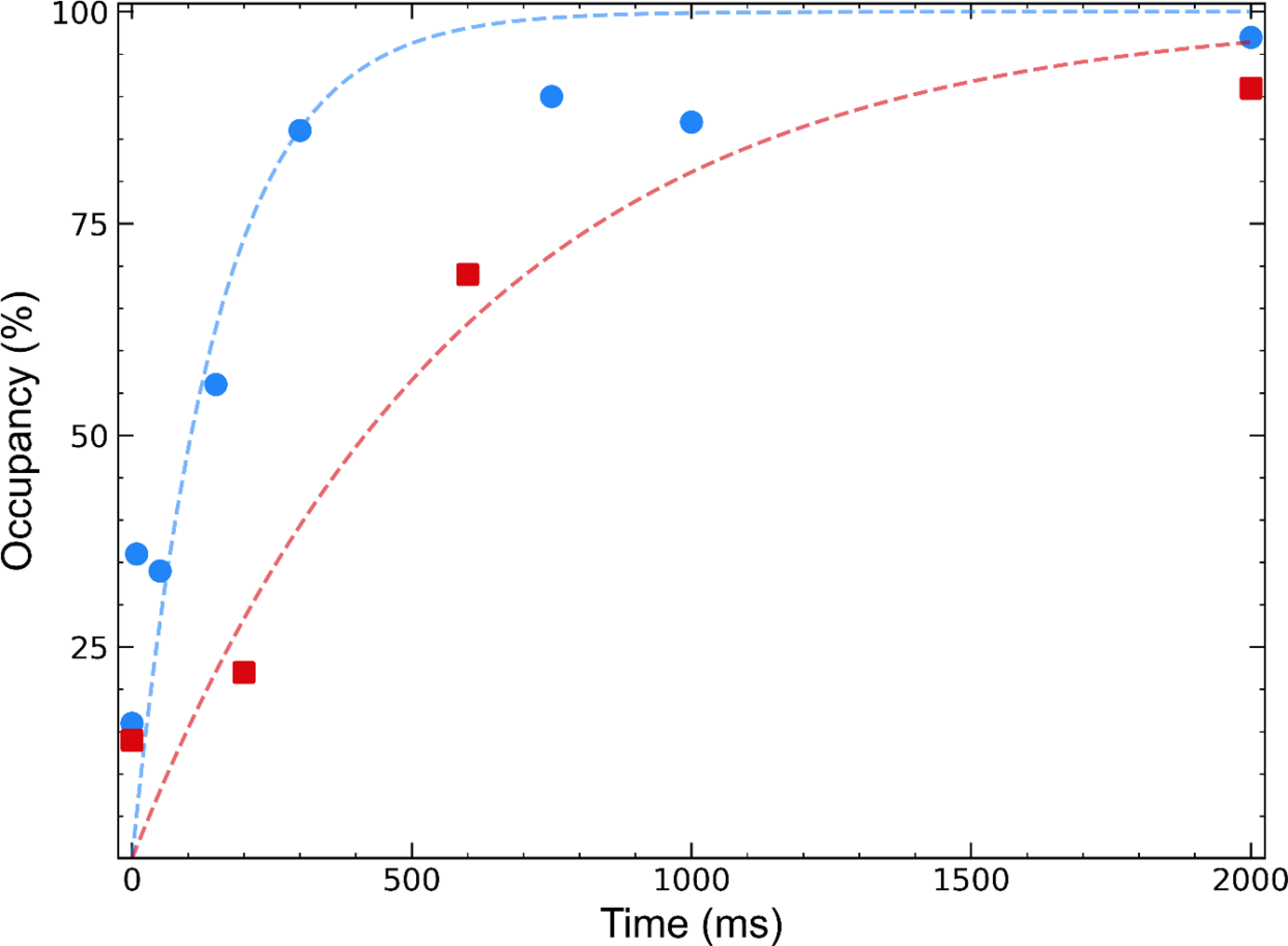
Comparison of refined occupancies of NAG1 in tetragonal lysozyme crystals obtained in the present mix-and-quench TR-X experiment (blue circles; Fig. 4 ▸) with those obtained in a previous drop-on-drop room-temperature serial crystallography experiment (Butryn *et al.*, 2021 ▸; red squares). Dotted lines represent simple exponential fits constrained to pass through 0% occupancy at *t* = 0 and 100% occupancy as *t* → ∞. The time constants are 152 ms (blue) and 602 ms (red). Nonzero occupancy at *t* = 0 is likely due to overlap of unmodelled atoms with the ligand position.

Fig. 5 ▸ shows the refined occupancy of NAG1 versus time determined using our mix-and-quench data (collected remotely using a standard cryocrystallography beamline) and using data from Butryn *et al.* (2021 ▸) [collected at room temperature following the deposition of two to six 120 pl drops of NAG1 solution onto 3 nl drops containing a slurry of smaller crystals (3–5 µm) supported on a moving polymer tape (Section S2)]. While both data sets yield monotonically increasing occupancies that saturate near 1 at long times, the timescale for saturation and thus the apparent diffusion and binding time for the drop-on-drop data is significantly longer (by a factor of roughly 4) than for our data, even though our crystals were larger. This difference may be due to a larger thickness of liquid surrounding each crystal in the crystal slurry through which ligand must diffuse, compared with our blotted crystals, and from a longer time to cover crystals with ligand solution (∼1 ms in our approach). In isomorphous difference maps comparing the *t* = 2000 ms structures from Butryn *et al.* (2021 ▸) and the present work (Supplementary Fig. S16), the only significant peaks are at disulfide bridges, which are broken due to radiation damage when using synchrotron sources and, as in our experiment, a single small crystal per time point.

The total time to prepare 64 sample supports (∼9 per time point for *t* > 0, where each support may hold multiple crystals) using our mix-and-quench instrument was 3 h. Remote X-ray data collection was completed in 6 h. Complete data sets were collected from 14 crystals, two for each time point, and data from only one of these was required to determine a refined structure. Consequently, only ∼25 min was required for sample mounting, raster scanning, crystal selection and data collection per time point. The total volume of ligand solution used was 7.5 µl × 64 sample supports = 480 µl, or an average of 60 µl per time point. Sufficient lysozyme crystals were obtained from a single 2 µl crystallization drop, containing ∼50 µg of protein, for each time point, for a total of 0.4 mg for eight time points.

Approximately 95% of prepared samples showed good deposition of ligand solution. For crystals that reliably diffract to high resolution, the number of sample supports prepared for each time point can safely be reduced by a factor of 3 and the preparation time per time point to ∼10 min. Together with improvements in loop design that have reduced the ligand solution required per sample support to 2 µl, this reduces the volume of ligand solution per time point to ∼6 µl.

In the drop-on-drop experiment (Butryn *et al.*, 2021 ▸), X-ray data were collected from ∼50 000 drops containing a total of ∼10 mg protein and ∼20 µl ligand solution per time point, requiring ∼30 min of XFEL time per time point.

## 5. Discussion

### 5.1. Mix-and-quench provides the easiest and most robust route to routine TR-X

The instrumentation and methods that we have developed provide a powerful and broadly applicable approach to time-resolved crystallography of chemically (versus optically) induced chemical and structural changes. Mix-and-quench TR-X now has multiple advantages over approaches where diffraction data are collected near room/biological temperature. Quenching decouples mixing and reaction from measurement. X-ray data can be collected at a later time and in a different place. Samples can be prepared in the home laboratory or in a central facility, cryogenically stored and then shipped to a synchrotron source days or months later. No special ‘chips’ are required for time-resolved data collection. Samples can be held on any thin-film or nylon-loop support, with excess surrounding liquid blotted away to ensure the shortest mixing times. Samples sufficient to obtain a complete time series of structures can be prepared in a few hours. Ligand-solution consumption is less than in other current methods, especially when dead volumes and volumes required to prime dispensed drop systems are considered. Switching between ligand solutions requires only cleaning/exchange of the loop and the use of a new pipette tip. No special hardware or protocols are required at the synchrotron. Data can be collected remotely using any standard high-throughput cryocrystallography beamline.

The time for data collection is not constrained by the reaction time and can be as long as necessary to make the optimal use of available crystals. Each crystal on a given sample support can be aligned in the X-ray beam and multiple frames of data collected from that crystal over a finite oscillation range, facilitating diffraction-pattern indexing. Reduced rates of radiation damage at cryogenic temperature allow ∼50–100 times more data to be collected from each crystal than at room/biological temperature. Complete data sets at each time point may often be collected using only one or a few crystals. Protein and crystal consumption per time point need be no more than in regular cryocrystallography and are orders of magnitude less than when using XFEL sources. The total cost of parts for our system, excluding machining costs, was ∼5500 USD, so that home-built or commercial versions should be affordable for most structural biology laboratories and central facilities.

Obtaining crystals with uniform shape and (small) size to obtain consistent diffusion/reaction times will often require time-consuming optimization. To avoid this, optical imaging and/or high-resolution X-ray rastering (after data collection) at two orthogonal angles can be used to estimate the size and shape of each crystal and this information can be used to model relative ligand diffusion times. For a desired time point, a subset of crystals with diffusion times that are short compared with that time point can then be selected for structure determination. *In crystallo* diffusion coefficients for ligands will seldom be known and may be factors of ∼2–100 smaller than in bulk solution (Geremia *et al.*, 2006 ▸; Tomadakis & Sotirchos, 1993 ▸). Processing subsets of crystals with closely similar sizes and shapes should yield more easily interpretable electron-density maps, and comparison between subsets should facilitate modelling to assess diffusion coefficients and the actual time point probed.

Together, these features of the mix-and-quench approach make time-resolved crystallography feasible using any biomolecular system that yields even small numbers of crystals that diffract to adequate resolution and in which at least some (if not all) aspects of *in vivo* activity can occur. Little additional crystallization effort will be required beyond that required to obtain a static structure. Our approach is ideally suited to routine home-laboratory-based TR-X and to sample- and time-efficient screening of potential TR-X targets, including for subsequent room/biological temperature study by serial TR-X at XFEL and synchrotron sources.<!?tpb=-6pt>

### 5.2. Optimization and further development

Several aspects of our current TR-X sample-preparation system can be improved.

The achievable time resolution is limited by the mixing and diffusion times and by the cooling time. Our plunge-through-film approach yields mixing times of the order of 1 ms (roughly the time for the active area of the sample support to traverse and be wetted by the ligand-solution film). This is comparable to those achievable by dispensing a single (nanolitre) drop and by microfluidic/coaxial mixers and is limited only by the thickness of the residual liquid on crystal surfaces. The residual liquid can be reduced through careful manual blotting or by using systems engineered for serial crystallography combining porous thin-film sample supports and backside suction applied in a humidified environment (Mehrabi *et al.*, 2020 ▸; Illava *et al.*, 2021 ▸). Cooling times are set by the crystal size, the thickness of the deposited ligand solution, the thickness of the supporting polymer film, and the cooling method.

The cooling time also determines the fidelity with which the room/biological temperature state at a reaction time point is captured. Comparisons of biomolecular structures determined at room and at cryogenic temperature, where typical cooling times have been one to two orders of magnitude larger than those achieved here, have generally found limited evidence of structural evolution during those cooling times, and this has long justified the near-exclusive use of cryogenic temperature data collection. However, widespread changes in the occupancy of minor side-chain rotamers (Fraser *et al.*, 2011 ▸; Keedy *et al.*, 2015 ▸), less frequent changes in major side-chain conformations, and less frequent changes in the conformations of mobile loops and flaps are observed. Cooling times in our system can be reduced below 2 ms (and quenching times below 1 ms) by reducing crystal plus ligand-solution film thickness towards 10 µm, which requires additional experimentation with sample-support and loop design. Cooling times can be further reduced to ∼1 ms by plunging at ∼3 m s^−1^ (a practical upper limit) and to ∼0.5 ms by plunging in LN_2_ cooled to 63 K. These cooling times may not capture room-temperature side-chain rotamer ensembles (which are modulated by overall thermal contraction of the crystal lattice; Juers & Matthews, 2004 ▸; Atakisi *et al.*, 2018 ▸), but should largely capture the conformations of the minority of loops, flaps and larger entities that show significant temperature sensitivity.

Ligand-solution consumption in our plunge-through-film deposition method of a few microlitres per time point is much less than in room-temperature TR-X methods using microfluidic or coaxial mixers and is likely to be small enough for the majority of TR-X studies. Even so, only ∼1% of the liquid dispensed into a loop is transferred to the sample support. Reducing the loop diameter towards 1 mm (which requires the reduction of sample wobble during high-speed plunges) and changes in loop design may allow ligand consumption to be reduced by an order of magnitude. Drop-on-demand dispensers can dispense the few nanolitres of ligand solution required to cover a typical thin-film sample support in a single drop. However, dispensers typically must be filled with a much larger volume (microlitres to millitres) to operate and may need priming and alignment requiring the firing of several drops before each drop deposition. Substantial additional solution may be required to calibrate dispenser parameters to obtain the desired dispensing performance. Whether dispensed-drop systems can yield a ligand solution-deposition performance sufficiently superior to that obtainable using the plunge-through-film method to justify their cost and complexity remains to be determined.

## 6. Related literature

The following references are cited in the supporting information for this article: Adams *et al.* (2010 ▸), Bhattacharjee *et al.* (2023 ▸, 2024 ▸), Costello (2006 ▸), Dandey *et al.* (2020 ▸), Deshchenya *et al.* (2022 ▸), Emsley *et al.* (2010 ▸), Hajdu *et al.* (2000 ▸), Kaledhonkar *et al.* (2019 ▸), Malla *et al.* (2023 ▸), Martin-Garcia *et al.* (2017 ▸), Moreau *et al.* (2019 ▸), Papasergi-Scott *et al.* (2024 ▸), Rampp *et al.* (2000 ▸), Schlichting & Chu (2000 ▸), Schmidt (2013 ▸), Torino *et al.* (2023 ▸), Vagin & Teplyakov (2010 ▸) and Winter *et al.* (2018 ▸).

## Supplementary Material

PDB reference: lysozyme–NAG1, 0 ms, 9mp3

PDB reference: 8 ms, 9mp4

PDB reference: 50 ms, 9mp5

PDB reference: 150 ms, 9mp6

PDB reference: 300 ms, 9mp7

PDB reference: 750 ms, 9mp8

PDB reference: 1000 ms, 9mp9

PDB reference: 2000 ms, 9mpa

Supporting information, including Supplementary Table and Figures, and captions to Supplementary Movies. DOI: 10.1107/S205225252500288X/min5001sup1.pdf

Supplementary Movie S1. DOI: 10.1107/S205225252500288X/min5001sup2.mov

Supplementary Movie S2. DOI: 10.1107/S205225252500288X/min5001sup3.mov
