# Supplementary material for: Instrumentation and methods for efficient time-resolved X-ray crystallography of biomolecular systems with sub-10 ms time resolution
Source: IUCrJ. 2025 Apr 25;12(Pt 3):372–83. doi: 10.1107/S205225252500288X (PMC12044851; doi:10.1107/S205225252500288X)
Supplement: Supplementary file 1 [file m-12-00372-sup1.pdf]

IUCrJ

Volume 12 (2025)

Supporting information for article:

Instrumentation and methods for efficient time-resolved X-ray crystallography of biomolecular systems with sub-10 ms time resolution

John A. Indergaard, Kashfia Mahmood, Leo Gabriel, Gary Zhong, Adam Lastovka, Matthew J. McLeod and Robert E. Thorne

S1. Mix-and-quench approaches to time-resolved crystallography

Mix-and-quench time-resolved crystallography experiments in the 1970s through mid-2000s examined systems where the timescales for reactions or conformational changes were minutes to hours (Schlichting & Chu, 2000), often requiring adjustment of solution conditions to slow down steps along the reaction coordinate and increase the lifetime of intermediate states (Schlichting & Chu, 2000; Moffat & Henderson, 1995; Hajdu *et al.*, 2000; Stoddard, 2001; Ding *et al.*, 2006). Accessible time resolutions were in part limited by the relatively weak and large x-ray beams then available, which required use of large crystals having long diffusion and cooling times. Both mixing and plunging in liquid nitrogen were performed manually. The minimum feasible time resolution with this approach is a few seconds.

In our initial plunge-through film mix-and-quench experiments in 2020 (Clinger *et al.*, 2021), the minimum time between reaction initiation – a well-defined event determined by the time when the crystal passed through ligand-containing film (with ~2 ms uncertainty) – and the start of cooling was 40 ms. This was adequate to observe a previously unobserved intermediate in the reaction of oxaloacetic acid with the enzyme phosophoenolpyruvate carboxykinase (PEPCK).

In 2023, Mehrabi et al. (Mehrabi *et al.*, 2023) described their “spitrobot” mix and quench system. Ligand-containing drops were dispensed using a commercial drop-on-demand dispenser onto crystals supported on ~400 µm diameter polyimide mesh supports. The sample support was then plunged using an electropneumatic piston into LN_2. Time-resolved structures of binding of glucose and 2,3-butanediol to xylose isomerase were determined with a quoted time resolution of 50 ms.

However, to cover the area of the support and ensure reaction initiation in all crystals present, between 200 and 250 drops of volume 150 pL were deposited on each sample, requiring a deposition time of between 40 and 50 ms. Translation of the sample from the deposition position to the LN_2 surface required ~40 ms, so the nominal time point (as defined here) varied between ~40 ms (for a crystal fortuitously hit by the first dispensed drop) and 90 ms, with an average of 65 ms and up to 50 ms of crystal-to-crystal variation. The cooling time between 273 and 200 K for a 13 µm lead thermocouple identical to that used here was 4.2 ms, 2.6 times longer than in our system. The volume of ligand solution dispensed on the sample support was at least 30 nl, 10 times larger than used here, on a support with only 4 times the area so that the average ligand solution thickness on the support was roughly 2 times larger than in our experiments. The expected cooling time from 273 to 200 K for the samples is thus between ~3 and 6 times longer than in our system, or between 12 and 24 ms. Although Mehrabi et al. reported “millisecond cryo-trapping by the spitrobot”, actual time points for reported x-ray structures were substantially larger than the quoted values, a factor of ~2 larger than were achieved in our first-generation plunge-through-film approach (Clinger *et al.*, 2021) and a factor of ~10 larger than reported here.

S2. Comparison with room-temperature time-resolved serial crystallography

The current record for time resolution in mixing-based time-resolved crystallography is held by mix-and-inject serial crystallography (MISC) performed at XFELs, where reaction initiation and data collection occur at the same (room) temperature. This approach has yielded reaction time points as short as 5 ms (Pandey *et al.*, 2021) and 3 ms (Malla *et al.*, 2023). These experiments used a microfluidic coaxial mixing injector (Calvey *et al.*, 2019) that mixed ~10×10×2 mm crystals with ligand solution to form a jet moving at ~26 m/s. The nominal time resolution is set by the time between the start of mixing – where the coaxial flows converge – and entry into the x-ray beam and so is roughly comparable to the nominal time resolution reported here. The 5 ms time point was measured at the European XFEL and required data collection (acquisition of indexable diffraction frames) from ~100,000 crystals. Since the duration of the x-ray pulse train at the European XFEL was only 0.4% of the time between pulse trains, and since the x-ray beam was roughly a factor of 10 smaller than the jet size, the number of crystals that flowed through the x-ray beam while during collection of data for the 5 ms time point was of order 10^8-10^9. The 3 ms time point was measured at the LCLS, which has a less favorable pulse structure leading to even less efficient utilization of crystals. Our mix-and-quench approach using comparably small crystals should yield comparable time resolution with ~10^7 fewer crystals (set by radiation dose limits per crystal).

An alternative and more crystal-efficient approach for mixing-based room-temperature time-resolved serial crystallography at XFELs and synchrotrons involves dispensing drops of ligand solution onto drops of microcrystal slurries held on a polymer tape (Butryn *et al.*, 2021) or onto crystals held on a sample support "chip" (Mehrabi *et al.*, 2020, 2019). The first method was used to study binding of NAG-1 ($GlcNAc_1$) to lysozyme (as here), and the second to study binding of NAG3 ($GlcNAc_3$) to lysozyme.

In the "drop-on-drop" method (Butryn *et al.*, 2021), two to six 120 pl drops of NAG-1 solution were deposited onto a 3 nl drop containing a slurry of 3-5 μm lysozyme crystals. x-ray data was collected at time points of 50 ms, 200 ms, 600 ms, and 2 s. For the 50 ms time point, x-ray data was collected from ~47,000 drops (with one XFEL pulse per drop), containing ~10 mg of protein. No appreciable occupancy was observed at 50 ms and the overall rise in occupancy was much slower than observed here. Even though the crystals were much smaller than those used here, the large volume of surrounding mother liquor and very limited mixing caused by impact of ligand solution drops on the slurry drops apparently led to a much slower rise in concentration within the crystals. In any mixing method where the crystals are surrounding by substantial liquid volumes, the minimum time resolution that can be meaningfully probed will be substantially longer and the connection of nominal "reaction times" to those that occur within the crystal may be uncertain.

In the "drop-onto-chip" method (Mehrabi *et al.*, 2019), a single 75 pl drop of ligand solution was dispensed onto each crystal and x-ray data collected at time delays of 50 ms, 100 ms, and 1 s. As in our approach, excess liquid was removed from crystals, so that the time for ligand diffusion through surrounding liquid was minimized. The 50 ms time point required data from ~40,000 crystals (~36,000 indexed diffraction patterns) with sizes comparable to those of crystals examined here. Ligand solution consumption of a few µl was comparable to that required here.

Compared with mix-and-quench approaches, room/biological temperature TR-X has the advantage of accurately capturing all conformational details accessed in the crystal, and its timepoints lack uncertainty associated with the cooling time. With cooling rates of ~10,000 K/s achieved here and larger rates (~40,000 K/s) achievable using smaller crystals and thinner liquid films, little conformational relaxation aside from that of side chain rotamers is likely, and quenching times of <2 ms (**Section S10**) add little to total timepoint uncertainty. With simpler and more flexible sample preparation, vastly lower sample consumption, vastly simpler data collection at synchrotron sources, and straightforward indexing, scaling and merging of data, mix-and-quench TR-X is the obvious choice for routine TR-X measurements and for large-scale screening of crystals and ligands for *in crystallo* activity. A mix-and-quench "pipeline" will allow more efficient and effective use of room-temperature serial TR-X at XFEL and synchrotron sources.

S3. Comparison with time-resolved single-particle cryo-EM

In time-resolved cryo-electron microscopy, protein and ligand solutions are mixed by sequentially spraying onto a grid (Berriman & Unwin, 1994; Dandey *et al.*, 2020; Klebl *et al.*, 2021) ("spray and mix") or are mixed and then sprayed/dispensed onto a grid (Bhattacharjee *et al.*, 2024; Kaledhonkar *et al.*, 2019; Bhattacharjee *et al.*, 2023; Torino *et al.*, 2023) ("mix and spray"); the grid thus prepared is then plunged into liquid ethane. A 1994 "spray and mix" experiment, in which drops were sprayed onto a plunging grid at a height of 4 mm above the liquid ethane surface (Berriman & Unwin, 1994), achieved a nominal time resolution of 5 ms.

Subsequent TR-cryoEM work (Berriman & Unwin, 1994; Chen *et al.*, 2015; Klebl *et al.*, 2021; Kontziampasis *et al.*, 2019; Walker *et al.*, 1995; Amann *et al.*, 2023) has not surpassed this time resolution. Most TR-cryoEM studies have had time resolutions >100 ms and often >1 s (Papasergi-Scott *et al.*, 2024). Resolutions typically achieved in TR cryo-EM reveal domain and main chain motions such as those critical in the function of biomolecular machines, many of which cannot occur in the constrained environment of crystals and which tend to occur on longer timescales, but often cannot reliably quantify side chain motions, changes in ligand poses, and evolution of products important in chemistry.

Achievable time resolution in TR-cryoEM is comparable with that achievable with our mix-and-quench approach to TR-X, for largely the same reasons. In TR-cryoEM, mixing and deposition times

can be 1-2 ms. The main limiting factor is set by the minimum height above the ethane surface at which the reaction can be initiated without significant precooling (at least several mm), and by practical limits on grid plunge speeds of 1-2 m/s. Precooling of ultra-thin cryoEM sample films in cold gas above the liquid ethane surface has not been adequately addressed in the cryo-EM literature. Cold gas can easily extend to a height of 5 mm or more, depending on the ethane fill level in the cup. Sample precooling will slow reactions and increase apparent reaction timescales.

In our approach to TR-X, the mixing time (i.e., the time to pass through the ligand solution film) is ~1 ms. With cold gas layer removal by our gas exchange manifold and rapid translation of the loop from a "warm" position, reaction initiation can occur within 2-3 mm of the LN_2 surface without precooling, and plunge speeds through the loop can be up to 2.5 m/s. Cooling/quenching times in LN_2 with our instrument can be 1 ms, within a factor of ~10 of what is currently achieved when cooling cryoEM grids in ethane (Costello, 2006). The main factors limiting time resolution are the time for ligand diffusion through liquid surrounding crystals and for diffusion into the crystals. The first limiting factor can be addressed by blotting or applying suction prior to plunging to reduce liquid thickness to a few µm, and by high-speed translation of the sample through the ligand solution film, which will thin residual surface liquid and drive rapid mixing. The second factor – diffusion within the crystal – can be addressed by using very thin crystals, and by using crystal forms with larger solvent channels and larger "free" (non-hydration) water fractions (Cvetkovic *et al.*, 2005) (greater than ~40%). Diffraction from crystals with thicknesses of ~1 µm can be reliably obtained at current state-of-the-art microfocus synchrotron beamlines and at XFEL sources. Even allowing for a factor of 10-50 reduction of *in crystallo* diffusion rates from their bulk values (Schmidt, 2013), *in crystallo* diffusion times of ~1 ms should be readily achievable.

S4. Design objectives for the mix and quench sample preparation system for time-resolved crystallography

The initial objectives for our instrument design, based in part on experience gained with the system described in Clinger et al. (Clinger *et al.*, 2021) were as follows.

- Accept crystals on standard crystallography loops/mounts and on serial crystallography supports held in standard goniometer-compatible bases (e.g., SPINE, ALS, SSRL) used at crystallography beamlines. This ensures compatibility with all available tools for high throughput cryocrystallography, including sample handling pucks, storage canes and dewars, shipping dewars, and beamline sample cryocooling and automounting systems.
- Achieve cooling times from ~273 K to 200 K (the protein-solvent glass transition temperature, below which protein function and most protein dynamics are quenched) and to ~150 K (below which the cryoprotected solvent vitrifies and its translational atomic motions are quenched) for crystals with minimum dimensions of about 25 µm of 5 ms or less. This requires cooling rates of ~15,000 K/s and 27,000 K/s, respectively.

- Plunge the sample at speeds of at least 1 m/s and less than about 4 m/s. This range has previously been shown to give large cooling rates without requiring excessive travel distances through the liquid nitrogen, without causing excessive splashing of liquid nitrogen, and without causing loss of crystals from the support.
- Achieve minimum time intervals between application of ligand solution and entry into liquid nitrogen of <10 ms. This requires that liquid application occur within 1 cm (at 1 m/s) of the liquid nitrogen surface.
- Ensure that the sample temperature remains at its initial temperature until it enters liquid nitrogen, so that reactions occur at constant temperature until quenching. This requires that all cold gas that normally is present above cold surfaces be removed before the sample plunge.
- Ensure that the ligand solution temperature remains at that of the initial sample temperature. For short time points, the ligand solution must be placed very close to (within millimeters of) the liquid nitrogen surface, where it will cool radiatively even if all cold gas has been removed.
- Allow easy loading of ligand-containing solution for each sample.
- Allow robust transfer of ligand-containing solution to the sample support, sufficient to cover all crystals on the support in a single, fast transfer step.
- Transfer ligand solution to the sample support without displacing crystals off the active (x-ray accessible) area of the support.
- Minimize the thickness of the deposited liquid on the sample support, subject to the constraint of providing good mixing and diffusion, to achieve the fastest cooling rates and smallest cooling/quenching times. Our target cooling times require liquid thicknesses of about 25 μm or less.
- Minimize the amount of ligand solution required per sample. Ligand solution consumption has two components: (1) the amount of liquid transferred to each sample; and (2) the minimum amount of liquid for a given ligand and concentration that must be prepared. For example, commercial drop dispensers may produce 50 pL drops but require a minimum ("dead") volume of ~1 mL to operate properly and to ensure that concentrations remain constant with time.
- After sample quenching, allow easy placement of cryocooled samples in cryocrystallography pucks and easy puck transfer to a storage dewar.
- Allow operation for at least 1 hour without significant frost accumulation in the liquid nitrogen or on instrument surfaces.
- Allow preparation of at least 20 samples (at different time points) per hour.

S5. User interface for mix and quench system control

The user interface for controlling the mix-and-quench system is written in Python. Figures S9 and S10 shows screenshots of the graphical user interface (GUI). The GUI has six tabs, as follows.

Manual plunge tab. This tab enables manual control of both the sample vertical translation stage and the ligand solution deposition stage as well as all peripheral devices including the lid and gas exchange manifold heaters, vacuum and dry N_2 make-up gas valves, the cryogenic LN_2 fill valve, and the sample stage brake, as well as temperature logging via thermocouples. Automated LN_2 level control is also possible via the GUI but awaits implementation of an LN_2 level sensing mechanism.

The plunge motion can be configured using the following controls:

- *Motion profile* – Selects pre-defined motion profiles (position and velocity vs time) or define arbitrary profiles.
- *Plunge Vac* – Sets the time interval between the opening of the vacuum and N_2 gas valves (for cold gas removal and replacement) and sample plunging.
- *Step Pause Plunge* – Used for longer reaction time points. The sample is translated a short distance through the loop and ligand solution film, paused, and then plunged into the LN_2.
- *Pre-plunge distance* – Sets the distance the sample should translate before pausing in step-pause-plunge (SPP) mode.
- *Pause time* – Sets time interval of pause in SPP mode.
- *Lift after plunge* – Lifts the ligand solution deposition stage a specified distance after completing a plunge to facilitate post-plunge sample handling.
- *Home after plunge* – Homes the sample translation stage immediately after plunging to facilitate rapid testing.
- *Autosave* - Automatically saves all sample-specific data after each plunge, such as position and velocity vs time, loop vertical position during ligand solution transfer to the sample, and other parameters set in the GUI.

The Manual plunge tab shows the position of both the sample vertical translation stage and the ligand deposition stage. The state of the sample stage motor and the system as a whole are also displayed.

Home moves the sample carriage to its initial position at the top of the plunge path. *Plunge* executes the plunge, accelerating the sample at 2g to a final plunge speed of 2 m/s (or some user defined speed). Position vs time and velocity vs position data is taken from the motor encoder and displayed after each plunge to allow for precise verification of each time point. *Step* allows movement in discrete increments along the plunge path. The position of the ligand deposition stage can also be stepped up and down. Other control selections allow power to the heater, heater controller, and Venturi vacuum valve to be toggled.

Auto plunge tab. The Auto Plunge tab facilitates sample preparation by automating the setup to achieving a given timepoint. The user selects a pre-defined motion profile and enters the desired time point, and can select options such as temperature logging, lifting the sample after plunging (to facilitate sample storage), or homing after plunging. When the user presses *Setup*, the software automatically

determines whether *Step-Pause-Plunge* mode is necessary, If SPP mode is necessary, the pause time is calculated, and the user is prompted to select the sample stage travel distance for the ligand deposition plunge. The ligand solution deposition stage is then positioned at the appropriate height. After setup is complete, pressing *Plunge* initiates the plunge.

Visualization tab. The visualization tab allows data files from every run to be viewed. Each data file contains the position-time data, the plunge configuration parameters, and other metadata about the selected plunge. If temperature logging was selected for a plunge, the temperature-time and temperature-position data are also stored in the file. The user can choose to show several interactive plots:

- Position vs time
- Velocity vs position
- Temperature vs time
- Temperature vs position

The user may also select to indicate the heights of the deposition loop and LN_2 surface on the graphs.

Drop dispensing tab. The drop dispensing tab allows reaction initiation by dispensing one or more drops of ligand solution using a commercial drop-on-demand dispenser onto a stationary sample support. The user selects the drop dispensing parameters (which determine the volume dispensed), the number of drops to be dispensed, and the time delay between dispensing and plunging of the sample. Dispensing on the fly – as the sample is plunged – is also possible.

Humidity control tab. This tab allows the humidity and temperature of an optional environmental chamber that encloses the plunge path to be set and monitored.

Utility tab. The utility tab allows several pre-defined routines to be run automatically. These include:

- *Gas layer measurement*: the cold gas layer thickness is measured by measuring the temperature along the plunge path near the LN_2 surface.
- *SPP timing characterization*: the *Step-Pause-Plunge* mode is timed over multiple repetitions to determine its repeatability and accuracy.
- *Command characterization*: determines the execution delay for sending motion commands to the motor controller for vertical sample translation, with 300 repetitions.
- *Pre plunge characterization*: the pre plunge motion is repeated continuously and measured to determine its repeatability and average profile. The results are stored in a standard profile file.
- *Plunge characterization*: the plunge motion is measured and repeated to determine its repeatability and average profile. The results are stored in a standard profile file and can be viewed.

- *Deposition stage characterization:* Coordinates vertical positions of the ligand deposition stage and sample stage to ensure proper ligand solution film placement during auto plunge setup.

S6. Plunge motion reproducibility

Side-to-side "wobble" of the plunge stage during its motion from its home position to the LN_2 surface was recorded by attaching an LED, masked so that only a small point of light was visible, to the sample wand and acquiring time-exposure images of its motion. Full side-to-side wobble amplitudes were ~0.7 mm when the sample was plunged at 1.8 m/s without braking, and ~1.4 mm when plunged with full braking. Wobble was repeatable from plunge to plunge, so that the sample could be reliably directed through a 2 mm diameter loop without hitting it.

The largest wobble amplitudes were observed near the start of motion and then when braking was applied. Although small, this wobble sets the minimum usable diameter of the ligand solution deposition loop and must be accounted for if reaction initiation is via drop-on-demand dispensing. Wobble during braking can be reduced by increasing the plunge depth through the LN_2 and thus reducing the braking force required to bring the sample to a stop. The plunge depth of the current instrument is only 6 cm, chosen to limit the amount of LN_2 held by the chamber to about 1 liter, and can easily be increased.

S7. Ligand solution deposition

Initial plunge-through-film experiments yielded irreproducible transfer of ligand-containing solution to the sample supports. In the experiments of Clinger et al. (Clinger *et al.*, 2021), roughly 10% of prepared samples had suitable liquid transfer. Observed behavior included minimal liquid transfer to the crystal-holding portion of the sample support, excessive liquid transfer to the neck of the support, displacement of crystals off the crystal-holding portion or off the support entirely, and film instability resulting in film popping before immersion of crystals.

Extensive experimentation using high frame rate imaging during liquid deposition and imaging of liquid deposited on cryocooled samples explored factors that may affect the quality of liquid transfer including the surface tension, viscosity and density of the ligand-containing liquid, the shape and design of the sample support, and the hydrophilicity/hydrophobicity of the sample support.

For ligand in CPA-free buffer — solutions with relatively large surface tension and small viscosity — held in 5 mm ID loops, the ligand solution film snapped and rapidly retracted toward the loop on first contact with the leading edge of the support, resulting in little liquid deposition. This issue became less pronounced with smaller diameter loops.

In practice, the ligand solution must contain CPAs to prevent ice crystallization during cryocooling, and these generally increase viscosity and decrease surface tension. We obtained good performance using 5% w/v PEG 4000 and 10% v/v PEG 400. Films were stable and did not "snap", but liquid transfer depended on the geometry of the sample support. Larger PEG concentrations increased viscosity and the amount of liquid transferred.

S8. Estimating volume of ligand solution transferred to a sample support.

To estimate the volume of ligand solution deposited onto a plunged sample, the sample was imaged at T=100 K using in-line optics at the synchrotron beamline before diffraction data is collected. Assuming the drop forms a spherical cap (a reasonable approximation based on observations), the volume V can be estimated from the diameter D of the profile of contact between ligand solution and crystal support surface and the height H of top of the ligand solution drop using

$$V = \frac{1}{6}\pi H \cdot \left(\frac{3}{4}D^2 + H^2\right)$$

Measurements of D and H were collected using the program ImageJ and scaled using the known calibration of the beamline imaging system.

S9. Relation between nominal and "actual" time points

Time points reported in previous time-resolved crystallography experiments are based on specific assumptions and often have very large uncertainties. For our plunge-through-film mix-and-quench approach, we have quoted nominal time points as the time interval from sample passage through the ligand solution film to first contact with the liquid nitrogen surface.

A better estimate for the time point probed in our experiments should account for (1) the time for ligand solution application onto the sample; (2) the time for ligand solution diffusion through any liquid initially present adjacent to the sample surface; (3) the time for ligand solution diffusion into the crystal; and (4) the time for the reaction to be quenched. Items 1-3 will decrease the estimate relative to our nominal time points, while item 4 will increase the estimate, and all will increase uncertainty in the estimate.

Time for ligand solution application. The ligand solution film thickness is <1 mm, the crystal containing portion of the sample support has a diameter of roughly 200 μm, and the sample speed during a direct plunge through the film and into liquid nitrogen is 1-2 m/s. High speed videos indicate that the sample is enveloped in liquid as soon as it enters the film. Uncertainty in the time of ligand solution application is thus <1 ms.

Time for diffusion through residual mother liquor on the crystal surface. Samples are carefully blotted to remove excess mother liquor or CPA solution prior to plunging. Interaction of residual surface liquid with the ligand solution film during high-speed plunging should rapidly thin and mix residual surface liquid, leaving a crystal size-dependent "boundary layer" of perhaps a few micrometers or less. The diffusion time for typical ligands through this liquid layer should be of order 1 ms (Schmidt, 2013).

Time for diffusion into the crystal. The time for diffusion to largely populate the interior of the crystal with ligand depends on crystal size and minimum dimension (thickness). In TR-X experiments, the diffusion coefficient is typically assumed equal to its value in bulk solution. Using Eq. 3 in Ref. (Schmidt, 2013) and a diffusion coefficient of 5×10^{-6} cm^2/s, for tetragonal lysozyme crystals of size

~10 μm the diffusion time is ~7 ms. This is likely an underestimate. Simple theory and simulation suggest that the diffusion coefficient may be reduced by the square of the solute-accessible volume fraction within the crystal (Geremia *et al.*, 2006; Tomadakis & Sotirchos, 1993). In tetragonal lysozyme, the total solvent volume fraction is ~40% and the solute accessible fraction (typically assumed to exclude the first hydration layer) may be half of that or even less, depending on the solute/ligand. This could increase diffusion times by a factor of ~10-25 (or more). On the other hand, ligand need only substantially populate a fraction of a crystal's volume (i.e., those regions closest to the crystal surface) to produce detectable signal in electron density maps. This may occur on a much shorter timescale than that for, e.g., total occupancy within the crystal volume to rise within 1/e = 63% of its final value, depending on crystal geometry. Average concentrations within a crystal volume may reach 10% of their final value in 1/6 to 1/8 the time required to reach 63% (Geremia *et al.*, 2006).

Time for quenching of binding / reaction. The cooling time can be estimated as the time for the sample temperature to drop from its initial temperature or from 273 K to ~200 K, where most protein dynamics and enzymatic activity cease, or to ~150 K, where internal non-hydration solvent vitrifies and solute/ligand diffusion ceases (Moreau *et al.*, 2019). For ~50 μm thick samples (crystal + ligand solution) in our experiment, the cooling time from 273 to 200 K is estimated (by power law scaling cooling times measured for 23 and 115 μm diameter thermocouples) to be roughly ~4.2 ms, corresponding to a cooling rate of ~17,000 K/s.

The cooling time determines the time for structural relaxation of the overall crystal+ligand system and thus the fidelity with which the structural state at the initial sample temperature is captured. The time for quenching of binding / reaction is in general shorter than the cooling time. It can be defined as the time to cool to a temperature below which subsequent diffusion and binding make a negligible contribution to observed occupancies. Diffusion coefficients drop with decreasing temperature, e.g., by a factor of 2 and 3.5 between 298 K and 273 K for sucrose in 10% and 50% w/w solutions, respectively (Rampp *et al.*, 2000; Deshchenya *et al.*, 2022). Binding and reaction rates also drop. Assuming a typical Q_{10} value of ~2, an initial sample temperature of 295 K, a cooling rate of 17,000 K/s, that concentration changes within the crystal due to diffusion during cooling can be neglected and that available binding sites do not become fully occupied, the total binding reaches 63% of its T=77 K value in ~0.9 ms (when the sample temperature is ~10 °C) and 90% in ~1.9 ms (when the sample temperature is -8 °C). The quenching time is thus <1/2 of the cooling time.

S10. Methods for TR-X study of binding of NAG-1 to lysozyme

Crystal growth. Lysozyme microcrystals were grown using a protocol similar to those previously described (Martin-Garcia *et al.*, 2017). Briefly, 50 μL of lysozyme at 50 mg/mL in 100 mM sodium acetate buffer pH 4.6 was added to 50 μL of 100 mM sodium acetate pH 4.6 + 20 % sodium chloride +

5 % PEG 4000. This mixture was immediately vortexed for 10 seconds. Crystals were grown over 1 hour and had typical dimensions of 10-15 µm.

TR-X sample preparation. Crystals were harvested in a home-made humidity tent set to provide 70-80% r.h. For each time point, 2 µL of resuspended microcrystal solution was pipetted onto a siliconized coverslip. MicroCrystal Mounts™ (MiTeGen, Ithaca, NY) were inserted into the solution to scoop up crystals and then dipped back into the solution to rinse off excess crystals. Excess liquid surrounding crystals was removed by touching the bottom of the mount to a paper wick. Crystals were then placed into a cryovial containing a wetted sponge to maintain humidity during transfer to sample plunge stage.

Ligand solution containing 226 mM NAG-1 in 100 mM sodium acetate pH 4.6, 17.5 % sodium chloride, 5 % PEG 4000, and 10 % PEG 400 was deposited onto the loop, and samples plunged through the loop (positioned at different heights for different time points) into LN_2.

Data collection. Apo diffraction data was collected at CHESS beamline 7B2 using 50 µm crystals, prepared in the same way as for time-resolved data but without NAG1 present. Time-resolved data was collected at the FMX beamline at NSLS-II using an X-ray beam with nominal size of 2.5 µm × 1.5 µm. Low dose rate rasters were used to locate individual high-diffracting crystals and 90° of oscillation data was collected per crystal. To estimate the size of each crystal, the beam was rastered with a 2.5 µm grid spacing after X-ray data collection was complete.

Data processing. All data was processed, scaled and merged using DIALS(Winter *et al.*, 2018). The molecular replacement solution was found using MOLREP (Vagin & Teplyakov, 2010) and PDB ID 7BHN as the starting model. Apo-data collected at CHESS was processed with DIALS. Refinement was completed using Phenix.refine (Adams *et al.*, 2010). Automatic water placement was done in Phenix.refine, and the structure was manually adjusted and refined with COOT (Emsley *et al.*, 2010). For occupancy refinements both B-factors and occupancies were simultaneously refined. Active site water occupancies (0 through 150 ms) were coupled to NAG-1 so that the sum of their occupancies equaled 1. Data from Butryn et al. (Butryn *et al.*, 2021) was re-refined in the same way for comparison. Final structure statistics are present in **Tables S1 and S2.**

Table S1 Scaling and refinement statistics for lysozyme – NAG1 structures for reaction time points between 0 and 150 ms.

	Lysozyme-NAG1 0 ms	Lysozyme-NAG1 8 ms	Lysozyme-NAG1 50 ms	Lysozyme-NAG1 150 ms
**PDB ID**	9MP3	9MP4	9MP5	9MP6
**Data Processing Statistics**				
**Wavelength (Å)**	0.9686	0.9793	0.9793	0.9793
**Resolution range (Å)**	77.6 - 1.30<br>(1.32 - 1.30)	78.35 -1.82<br>(1.85 - 1.82)	37.52 - 1.97<br>(2.00 - 1.97)	77.83 - 2.22<br>(2.26 - 2.22)
**Space group**	P 43 21 2	P 43 21 2	P 43 21 2	P 43 21 2
**Unit cell (Å, °)**	77.462 77.462<br>38.288 90 90 90	78.277 78.277<br>37.626 90 90 90	78.27 78.27<br>37.516 90 90 90	77.778 77.778<br>38.175 90 90 90
**Total reflections**	173031 (5312)	43131 (2020)	55686 (2793)	39236 (1956)
**Unique reflections**	2682(1367)	9603 (484)	8501 (421)	6123 (307)
**Multiplicity**	6.45 (3.89)	4.49 (4.17)	6.55 (6.63)	6.41 (6.37)
**Completeness (%)**	91.74 (93.44)	87.59 (90.30)	98.12 (100.00)	99.33 (100.00)
**Mean I/sigma(I)**	18.9 (1.2)	16.1 (2.1)	6.6 (1.1)	5.1 (1.7)
**Wilson B-factor ($Å^2$)**	16.25	17.7	23.05	24.64
**R-merge**	0.048 (0.442)	0.143 (0.687)	0.200 (1.048)	0.292 (1.139)
**R-meas**	0.052 (0.509)	0.162 (0.783)	0.217 (1.136)	0.318 (1.236)
**R-pim**	0.019 (0.245)	0.073 (0.367)	0.081 (0.421)	0.123 (0.472)
**CC1/2**	0.999 (0.844)	0.991 (0.663)	0.986 (0.558)	0.735 (0.554)
**Refinement Statistics**				
**Resolution range**	54.7 - 1.30<br>(1.35 - 1.30)	55.35-1.82<br>(2.08-1.82)	33.83-1.97<br>(2.25-1.97)	38.89-2.22<br>(2.80-2.22)
**Reflections used**			8451 (2776)	6083 (3000)
**in refinement**	26786 (2722)	9558 (3205)		
**for R-free**	1357 (137)	465 (139)	414 (133)	288 (145)
**R-work**	0.1794 (0.2541)	0.1687 (0.1873)	0.1909 (0.2192)	0.1929 (0.2236)
**R-free**	0.2243 (0.2695)	0.2215 (0.2849)	0.2573 (0.3126)	0.2516 (0.3027)
**Number of**				
**non-hydrogen atoms**	1225	1197	1125	1102
**macromolecules**	1027	1021	1000	1000
**ligands**	5	20	20	20
**solvent**	193	156	105	82
**Protein residues**	129	129	129	129
**RMS(bonds) (Å)**	0.005	0.005	0.006	0.002
**RMS(angles) (°)**	0.75	0.72	0.86	0.5
**Ramachandran**				
**favored (%)**	98.43	99.21	98.43	99.21
**allowed (%)**	1.57	0.79	1.57	0.79
**outliers (%)**	0	0	0	0
**Rotamer outliers (%)**	0.92	0	0.95	0.95
**Clashscore**	4.45	4.91	3.01	5.52
**Average B-factor ($Å^2$)**	20.35	19.88	24.02	29.11
**macromolecules**	18.66	18.65	23.46	28.73
**ligands**	20.41	23.32	26.74	33.93
**solvent**	29.32	27.54	28.91	32.65

Table S2 Scaling and refinement statistics for lysozyme – NAG1 structures for reaction time points between 300 ms and 2000 ms.

	Lysozyme-NAG1 300 ms	Lysozyme-NAG1 750 ms	Lysozyme-NAG1 1000 ms	Lysozyme-NAG1 2000 ms
**Data Processing Statistics**				
**PDB ID**	9MP7	9MP8	9MP9	9MPA
**Wavelength (Å)**	0.9793	0.9793	0.9793	0.9793
**Resolution range (Å)**	37.81-2.15 (2.19-2.15)	38.21-1.83 (1.86-1.83)	77.95-1.90 (1.93-1.90)	55.03-1.85 (1.88-1.85)
**Space group**	P 43 21 2	P 43 21 2	P 43 21 2	P 43 21 2
**Unit cell (Å, °)**	78.069 78.069 37.806 90 90 90	77.829 77.829 38.197 90 90 90	77.889 77.889 37.769 90 90 90	77.821 77.821 37.745 90 90 90
**Total reflections**	42608 (2143)	70194 (3245)	62060 (3149)	67327 (3498)
**Unique reflections**	6255 (317)	10546 (517)	9203 (456)	10351 (518)
**Multiplicity**	6.81 (6.76)	6.66 (6.28)	6.74 (6.91)	6.50 (6.75)
**Completeness (%)**	92.56 (94.63)	97.41 (98.10)	95.84 (100.00)	99.82 (100.00)
**Mean I/sigma(I)**	7.1 (1.4)	9.0 (1.0)	7.2 (0.9)	8.7 (1.1)
**Wilson B-factor (Å^2)**	26.87	22.47	24.21	23.42
**R-merge**	0.238 (1.231)	0.156 (1.328)	0.184 (1.136)	0.165 (1.273)
**R-meas**	0.257 (1.339)	0.169 (1.442)	0.198 (1.225)	0.179 (1.379)
**R-pim**	0.094 (0.501)	0.062 (0.543)	0.072 (0.445)	0.992 (0.595)
**CC1/2**	0.975 (0.486)	0.992 (0.568)	0.992 (0.663)	0.992 (0.595)
**Refinement Statistics**				
**Resolution range (Å)**	34.03-2.15 (2.71-2.15)	34.29-1.83 (2.01-1.83)	38.94-1.90 (2.09-1.90)	33.96-1.85 (1.92-1.85)
**Reflections used for R-free**	309 (147)	524 (51)	526 (122)	530 (54)
**R-work**	0.1938 (0.2295)	0.1758 (0.2697)	0.1784 (0.2290)	0.1841 (0.2547)
**R-free**	0.2449 (0.3053)	0.2085 (0.3383)	0.2201 (0.3224)	0.2387 (0.2963)
**Number of non-hydrogen atoms**	1089	1142	1134	1111
**macromolecules**	1006	1012	1008	1000
**ligands**	20	20	20	20
**solvent**	63	110	106	91
**Protein residues**	129	129	129	129
**RMS(bonds) (Å)**	0.003	0.006	0.006	0.006
**RMS(angles) (°)**	0.6	0.8	0.73	0.82
**Ramachandran favored (%)**	98.43	98.43	98.43	00.21
**allowed (%)**	1.57	1.57	1.57	0.79
**outliers (%)**	0	0	0	0
**Rotamer outliers (%)**	0	0	0	0
**Clashscore**	2.5	2.48	4.49	2.01
**Average B-factor (Å^2)**	30.19	25.97	27.68	25.1
**macromolecules**	29.91	25.29	27.02	24.58
**ligands**	32.99	23.23	26.27	23.39
**solvent**	33.77	32.68	34.18	31.16

A

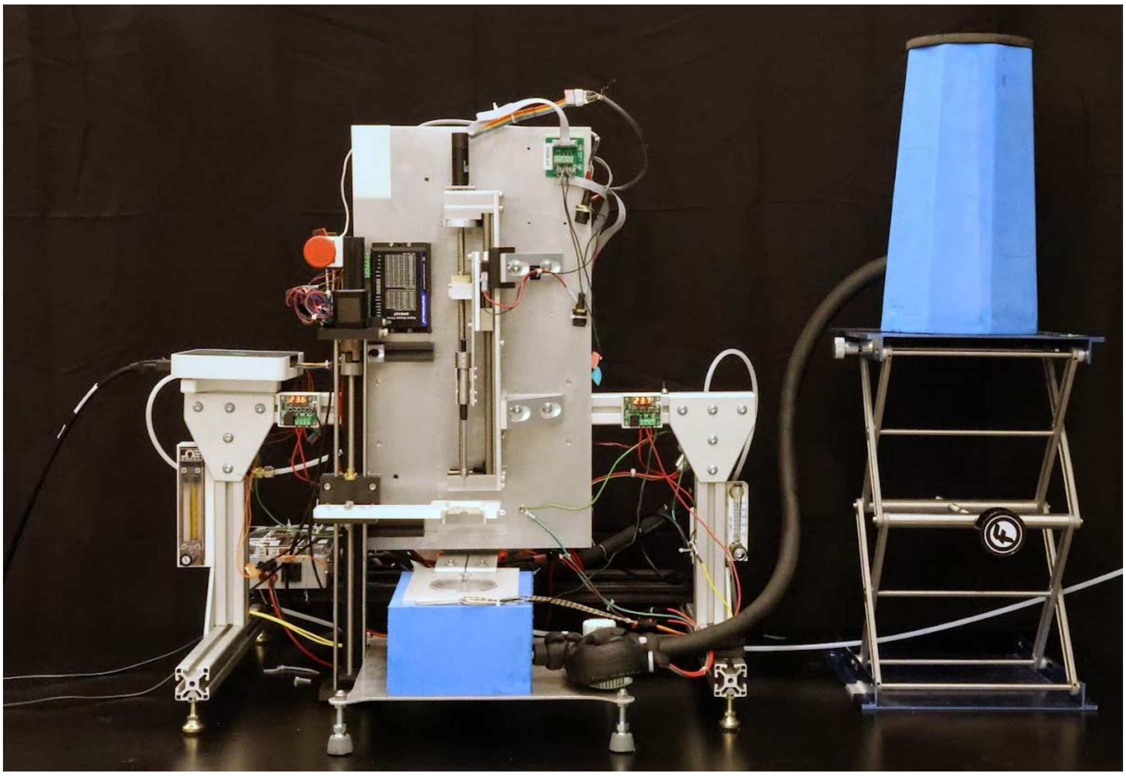

B

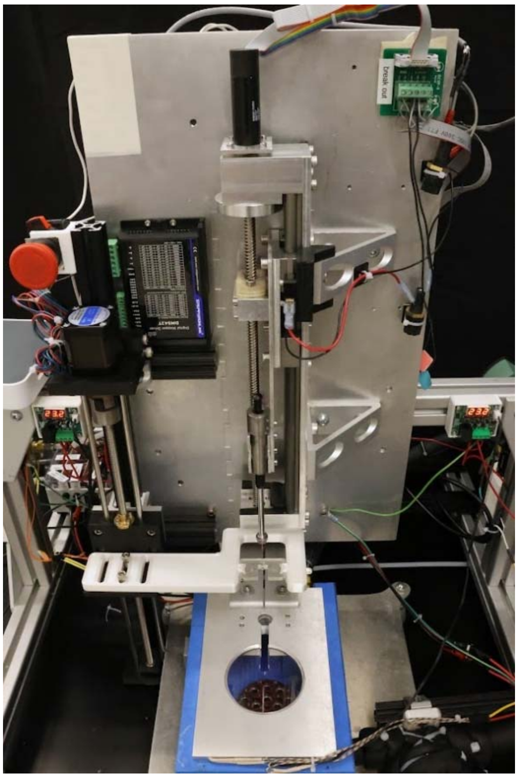

Figure S1 Photographs of the Mix-and-Quench sample preparation workstation for time-resolved crystallography (TR-X). **A** Complete system excluding control electronics and computer. **B** Close-up showing plunge and deposition stages, and liquid nitrogen chamber holding a Unipuck, with a heated lid and transparent rotating window for viewing the puck and depositing samples into it.

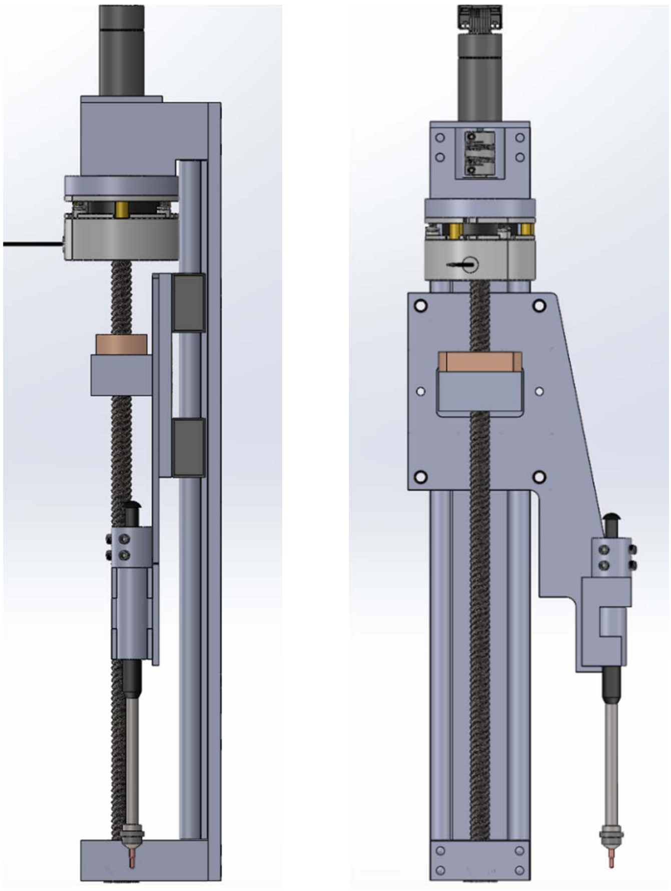

A **B**

Figure S2 **A** Front and **B** side views of the high-speed vertical translation stage for sample translations. A servo motor drives a lead screw, which drives the sample carriage up and down. Two guide rails minimize wobble and maximize reproducibility of the sample carriage motion. The sample can be slowed toward the end of the plunge by the motor and stopped by applying an electromagnetic brake.

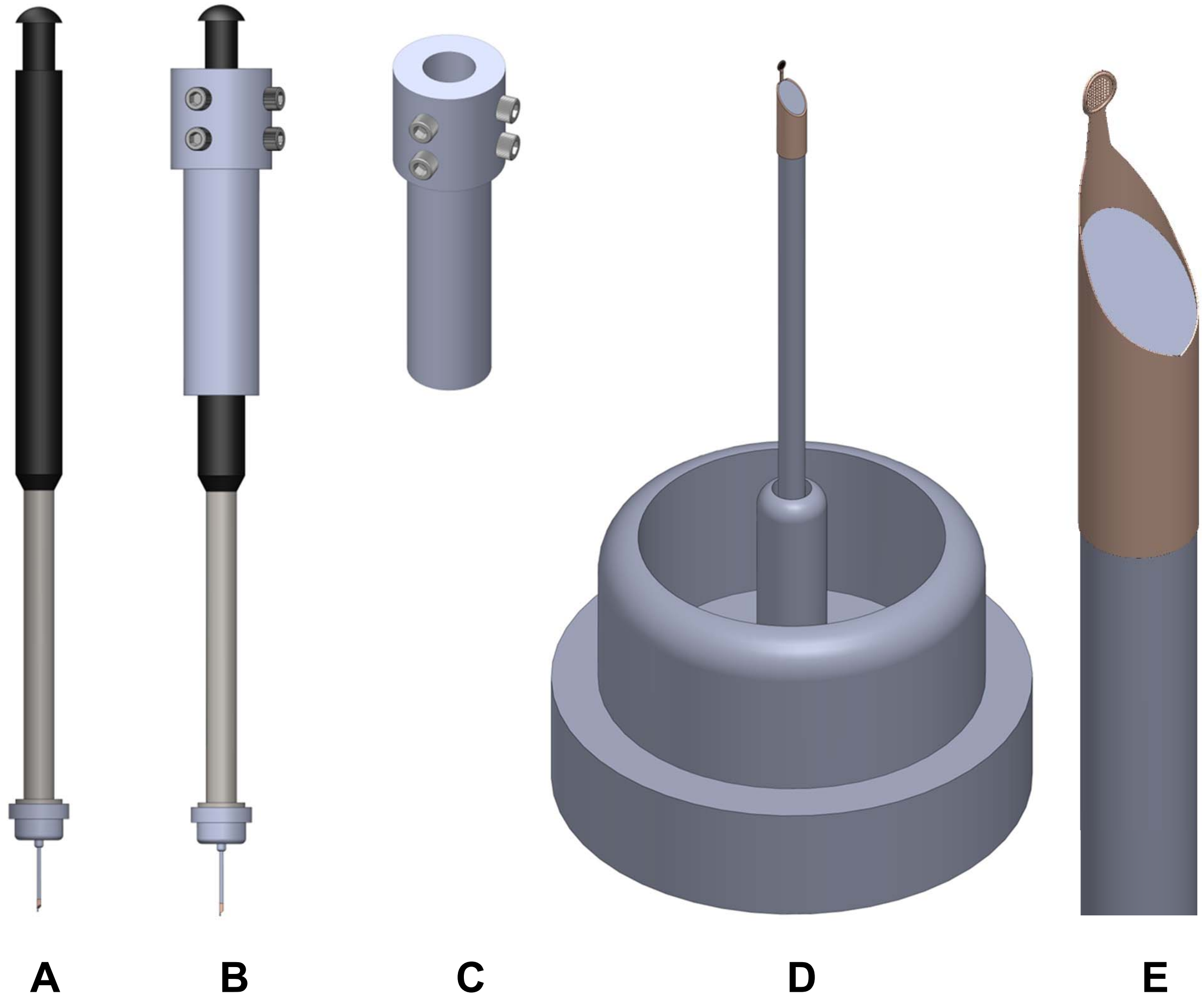

Figure S3 **A** Standard wand for cryocrystallography, with goniometer base and sample support. **B**, **C** A magnetic steel adapter holds the wand and inserts into the sample carriage (**Figure S2**) where it is held using magnets. **D** A microfabricated sample support (MicroCrystal Mount™) on a steel pin is held in a standard magnetic steel goniometer base (SPINE style). The goniometer base is magnetically held at the end of the wand in A. **E** Close-up of the sample support.

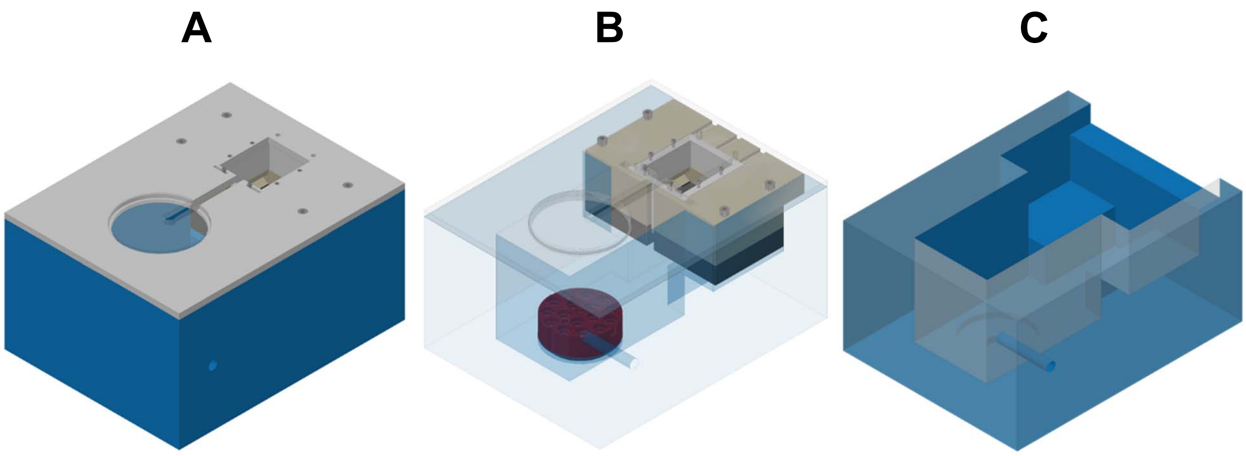

Figure S4 **A** Sample cooling and storage reservoir. A lid rests on the reservoir base. The main body of the lid is machined from aluminum and includes two cartridge heaters and a thermistor, allowing the lid temperature to be measured and controlled. The lid has a hole for the plunge bore and a slot connecting the plunge bore to the main portion of the reservoir. A circular window rotates freely within the lid and also has a slot. The slots allow a sample on a magnetic wand to be transferred from the plunge bore through the channel and into the main portion of the chamber, and then rotated and translated into position above a sample receptacle for storage, all while remaining immersed in LN_2. **B** The gas exchange manifold (GEM) attaches to the underside of the lid. A Unipuck is inserted by removing the round window. **C** The reservoir base is machined from a single piece of high-density polyurethane foam (used in commercial LN_2 dewars). The circular recess and alignment pin hold and orient a standard 16-sample crystallography Unipuck, used for sample storage, shipping, and automated handling at the x-ray source. The reservoir is filled with LN_2 via a tube in its side. The plunge channel is defined by two cuboids.

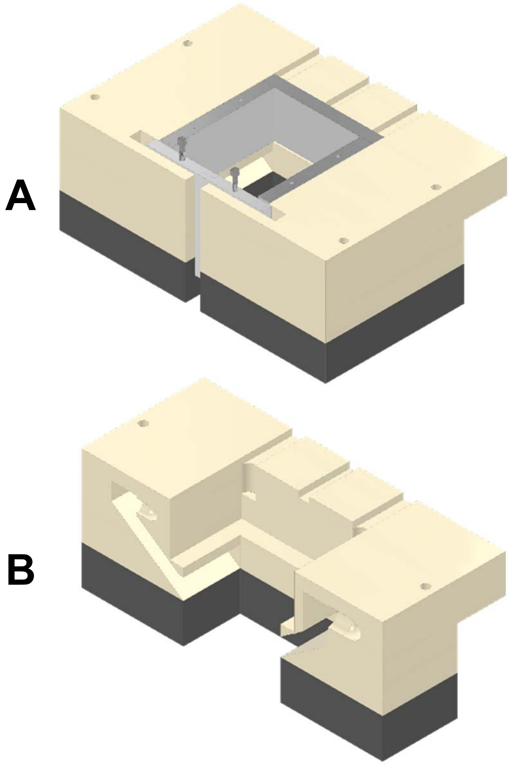

Figure S5 Gas exchange manifold (GEM). **A** The gas exchange manifold has a vertical plunge bore and a horizontal channel for passage of a sample-holding wand into the main portion of the sample cooling reservoir. The plunge bore is sealed off from the horizontal channel during cooling by a removable door. **B** Two downward sloping channels intersect the plunge bore. Immediately before a sample plunge, vacuum / suction is applied to one and dry ambient temperature N_2 gas to the other to remove cold gas present above the LN_2 surface and prevent precooling of the sample and ligand solution.

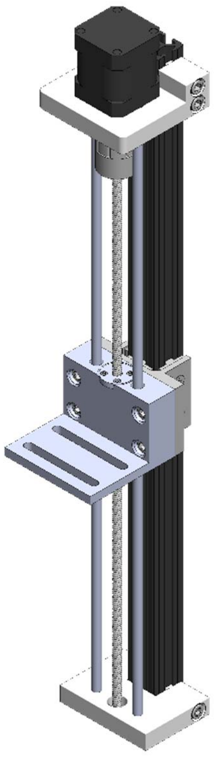

Figure S6 Ligand solution deposition stage. A stepper motor drives a lead screw that in turn drives the deposition carriage up and down. Two rails minimize the side-to-side motion of the carriage. Platforms holding different solution deposition mechanisms attach to the deposition carriage.

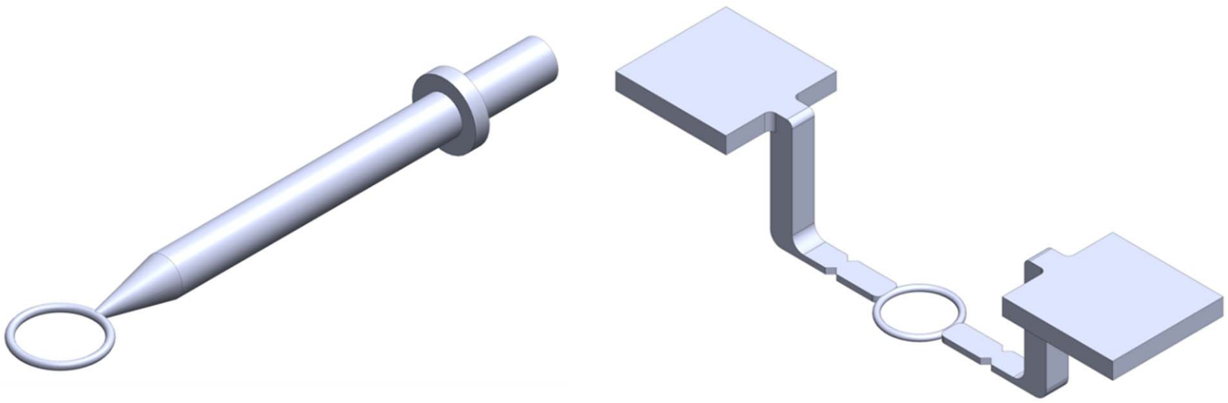

A **B**

Figure S7 Break-away loops for suspending ligand solution films on the sample's plunge path, used in early experiments. The loop ID is 3 mm. **A** A loop and support member that insert into a platform attached to the ligand solution deposition stage or into a rigid vertical member having an array of holes at different heights above the LN_2 surface. Upon impact of the large diameter portion of the goniometer base, the loop breaks off the support member at a constriction adjacent the loop, allowing the sample, base and wand to continue their downward travel into the LN_2. **B** A loop and support member that drop into a platform attached to the ligand solution deposition stage. The lower, loop-holding portion fits within the plunge bore of the gas exchange manifold and allows the loop to be lowered to within ~2 mm of the LN_2 surface.

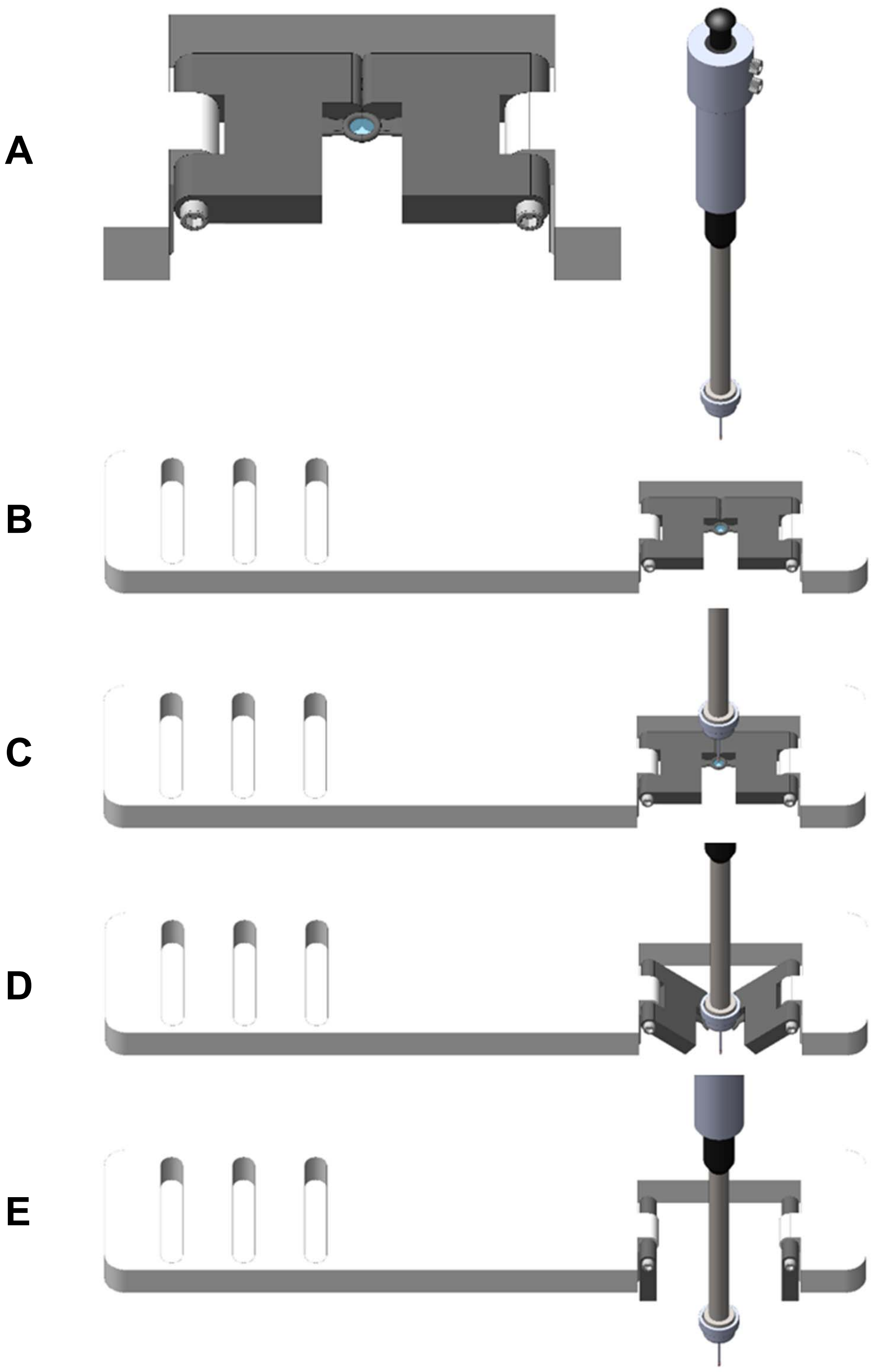

Figure S8 Plunge-through-film ligand solution deposition mechanism. **A** Roughly 2 µL of ligand solution is loaded into a 2 mm diameter loop. The loop is formed from two halves, each attached to a "door", and the doors are held in the closed position using magnets. **B** The doors attach to the ligand solution deposition platform, which in turn attaches to the deposition stage. Samples are held on a crystallography support (MicroCrystal Mount #1, MiTeGen) inserted into a goniometer base, and the base is held by a magnetic wand. **C** Liquid is transferred onto the samples and supporting film as they pass through the ligand solution film spanning the loop. **D** Impact of the goniometer base with the

doors pushes them downward. **E** With the doors open, the base and wand to continue to the LN_2. **Figure 2** shows a modified version of this mechanism that allows the loop to be placed within 2 mm of the LN_2 surface, corresponding to a ~1 ms time between ligand solution application and the start of cooling.

A

B

C

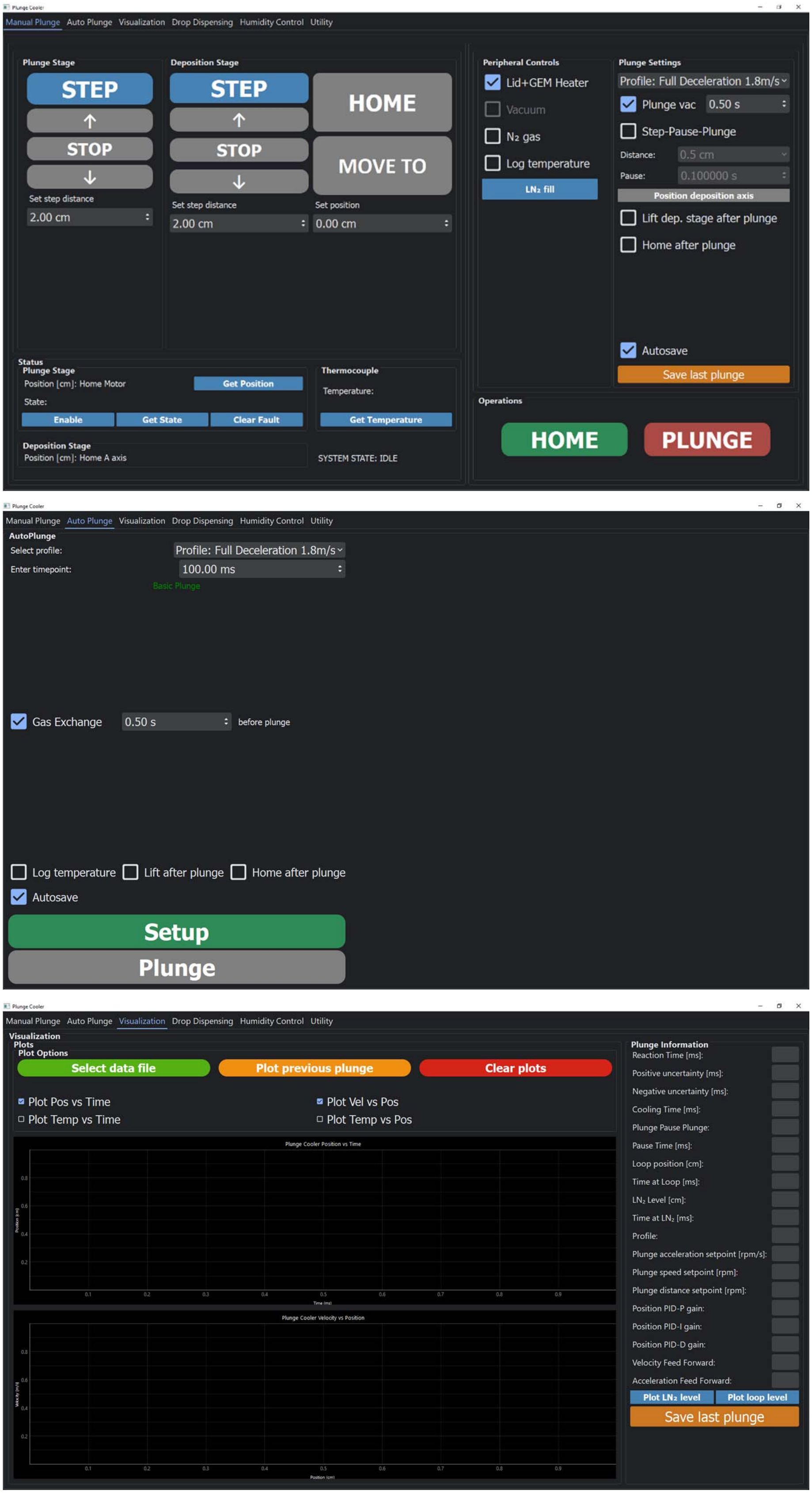

Figure S9 Screen shots of the first three tabs of the GUI used to control the system of **Fig. 1**. **A** *Manual Plunge*, **B** *Auto Plunge*, **C** *Visualization*.

A

B

C

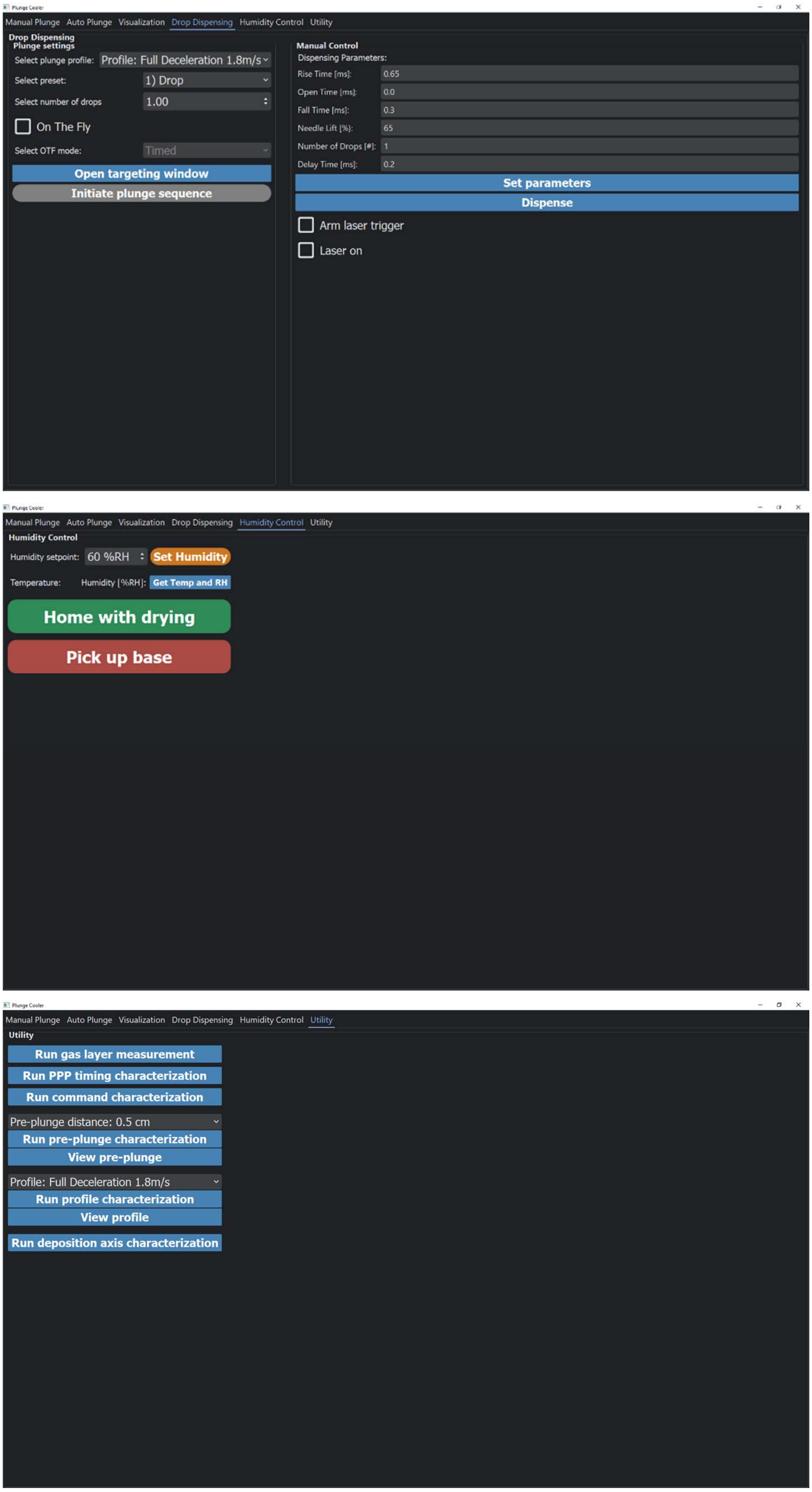

Figure S10 Screen shots of the last three tabs of the GUI used to control the system of Fig. 1. **A** *Drop Dispensing*, **B** *Humidity Control*, and **C** *Utility*.

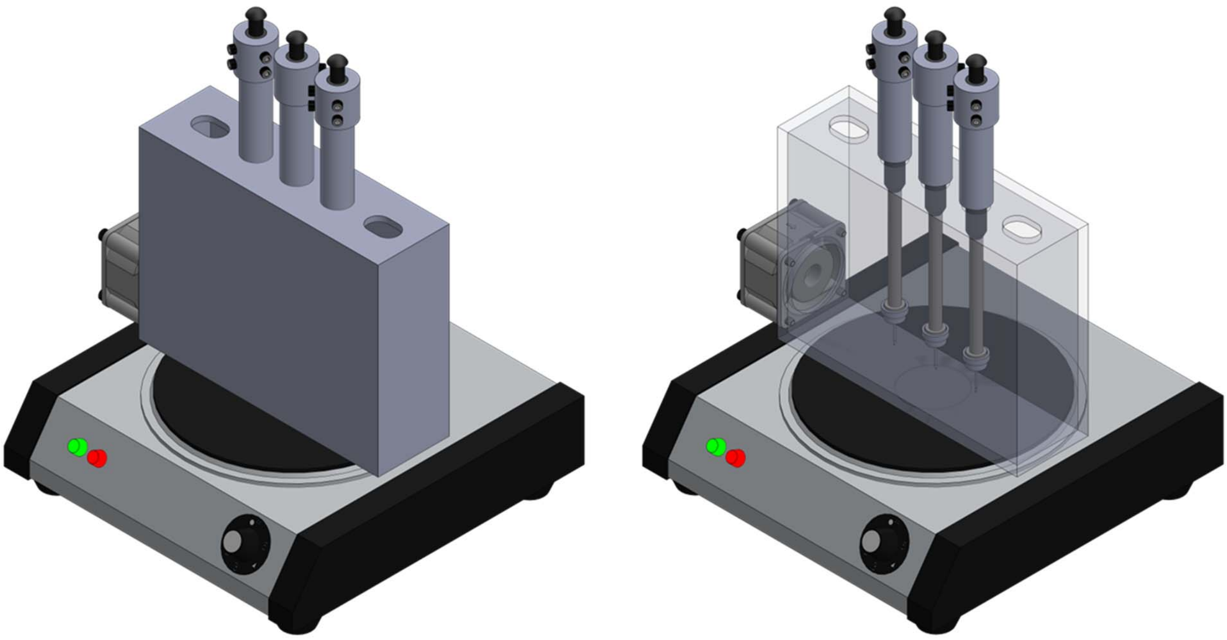

Figure S11 Warming station for warming and drying sample wands after each plunge.

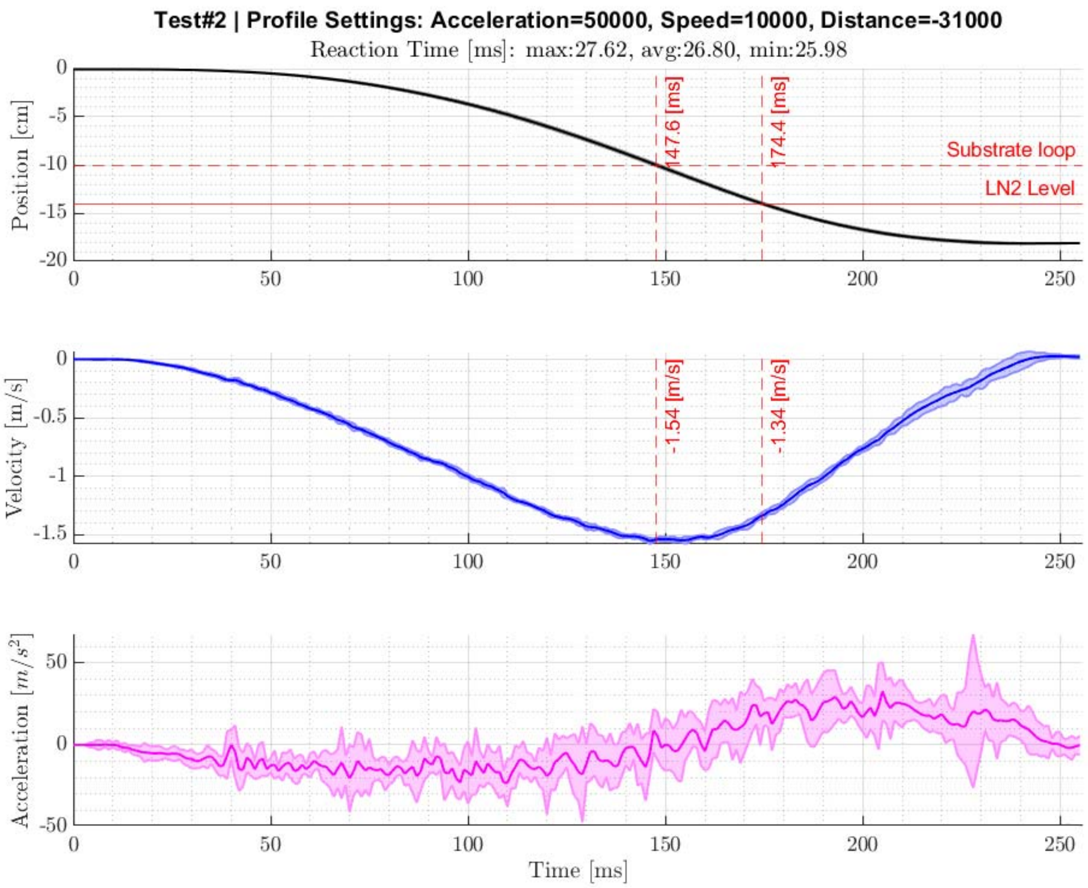

Figure S12 Reproducibility of sample position, velocity, and acceleration for 10 consecutive plunges with a maximum speed of 1.5 m/s and motor-controlled deceleration in the LN_2. In this example, the average sample speed on entering the LN_2 is 1.34 m/s. Sample position vs time determined using the plunge stage motor encoder. Overall reproducibility of position and velocity is excellent. The time interval from reaction initiation/passage through the loop and entry into LN_2 has an average value of 26.8 ms and varies between 26.0 and 27.6 ms over the 10 plunges.

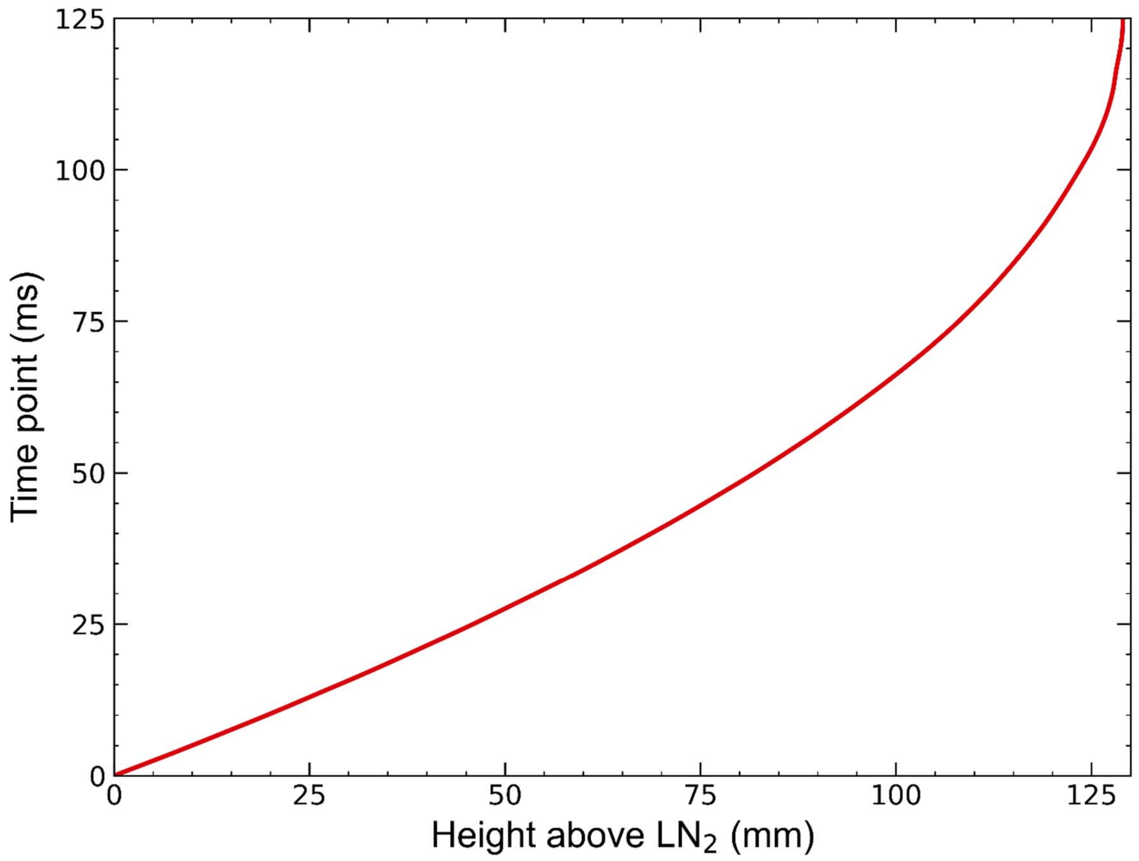

Figure S13 Nominal reaction time (time between ligand deposition on the sample and sample entry into LN_2) versus the height of the deposition loop above the LN_2, for the plunge velocity profile in **Figure S12**. The control software calculates and stores similar curves for other programmed plunge velocity profiles.

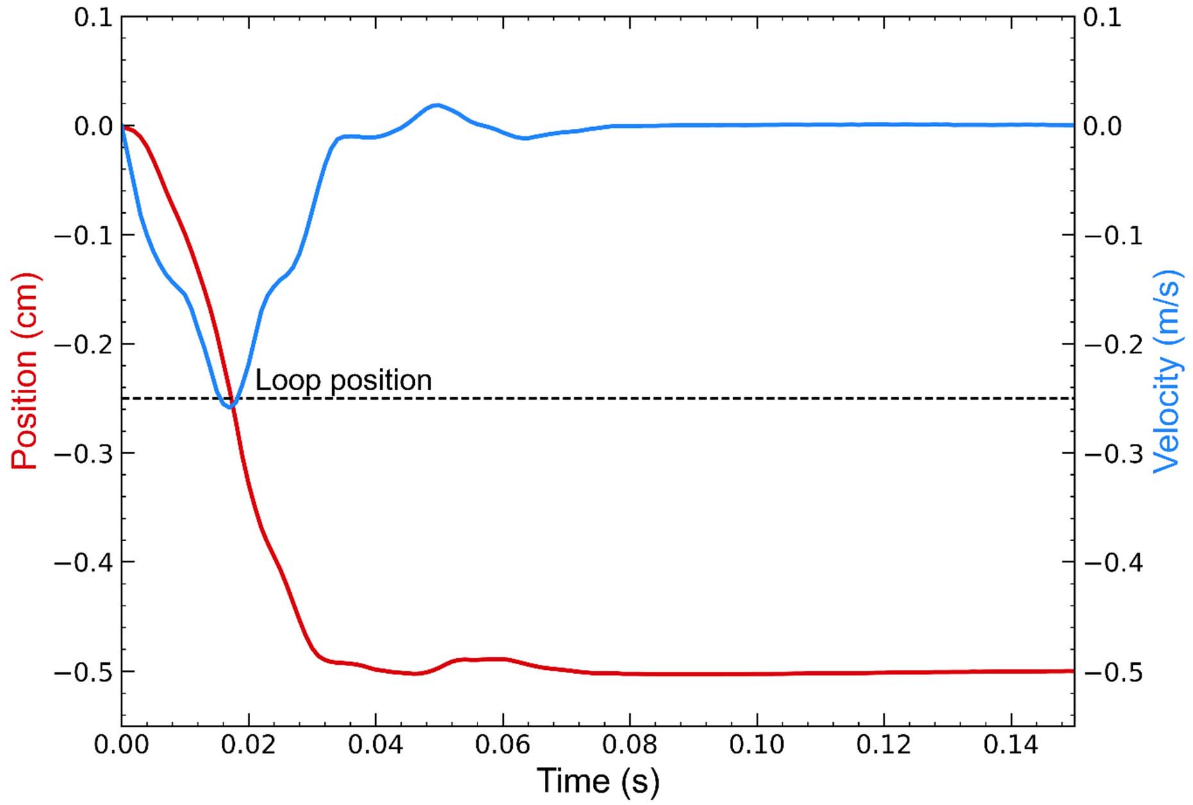

Figure S14 Sample position and velocity versus time during a sample "step" of 0.5 cm, used to translate the sample through the ligand-solution-containing film to initiate the reaction in a "*step-pause-plunge*" sequence is used to initiate and quench reactions for reaction times greater than 150 ms. The "step" is completed in ~30 ms.

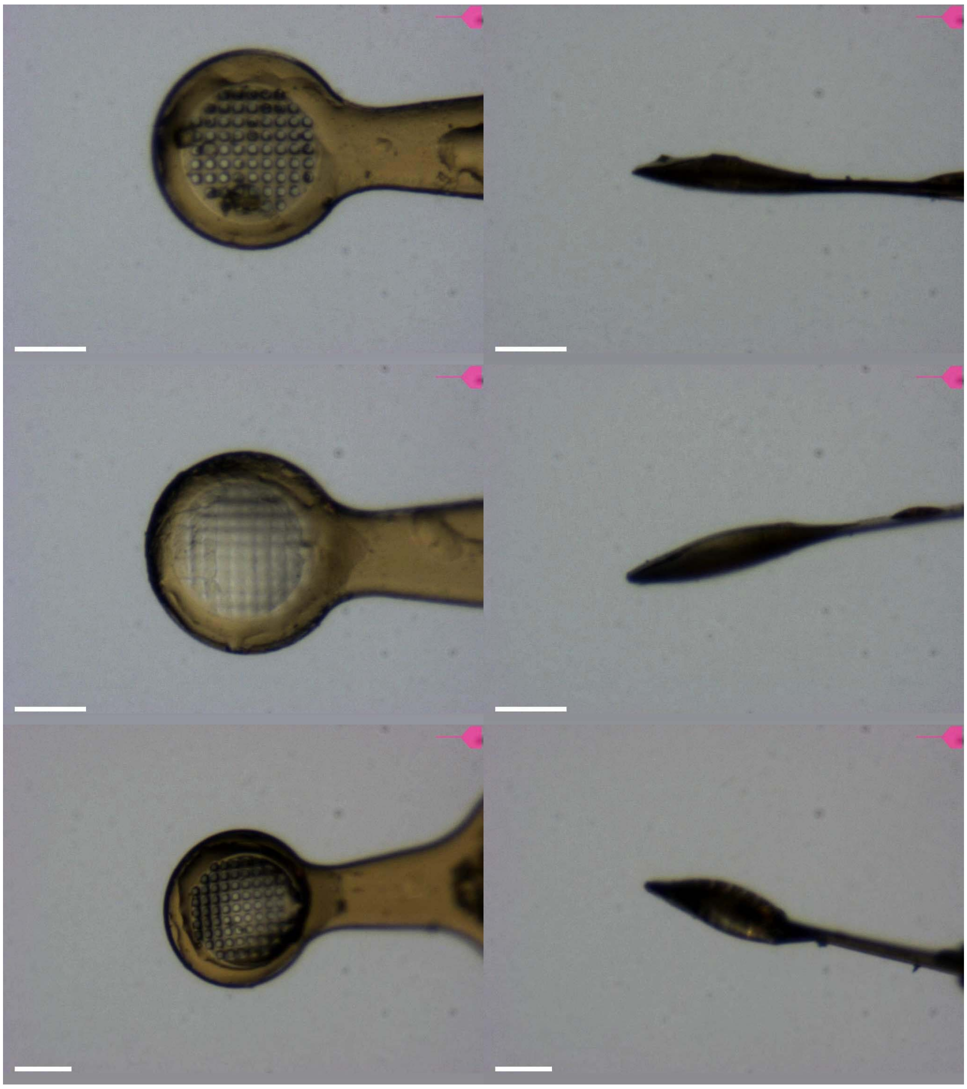

Figure S15 Example images of ligand solution deposition on crystal-holding sample supports following plunging through 3 mm ID loops loaded with 7.5 µL of solution. Images were recorded at T=100 K at the FMX beamline at NSLS-II during collection of data that yielded the structures in Fig. 4. Nearly all liquid surrounding crystals is blotted away before ligand solution deposition, so the liquid present was transferred from the thin film spanning the deposition loop. The thickness of liquid deposited was typically around 60 µm. Scale bars: 100 µm. Current loops have 2 mm ID and are loaded with 1-2 µL of solution.

A

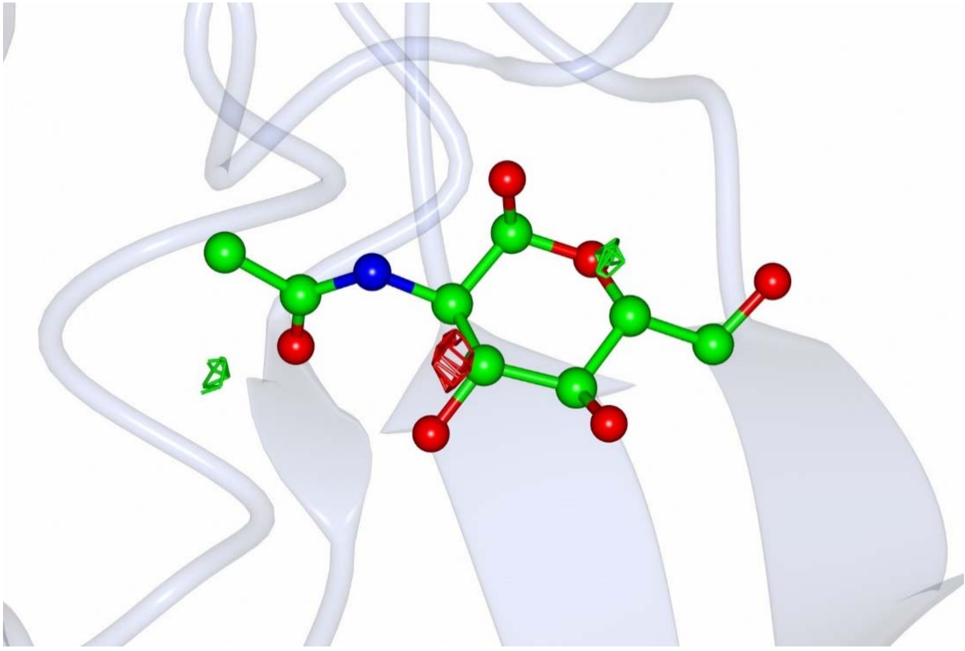

B

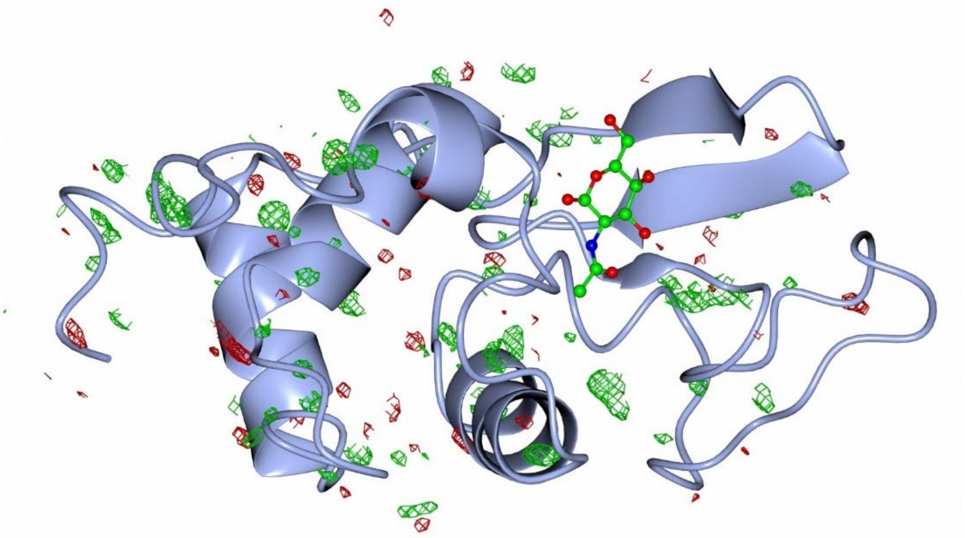

Figure S16 t = 2000 ms isomorphous differences between time-resolved structures determined using drop-on-drop room temperature crystallography with an XFEL source (from Ref. 8 in the main text) and millisecond mix-and-quench crystallography with a synchrotron source (present data). **A** Local (rendered at 2.5 σ) and **B** global (3.0 σ) isomorphous difference maps obtained by subtracting the present mix-and-quench data from the previous serial drop-on-drop data (Butryn *et al.*, 2021). The most significant positive peaks in the global representation are at disulfide bridges, which are intact in the "radiation damage free" XFEL data. The mix-and-quench structure was determined using a single (1) crystal of size 10-15 μm at T=100 K, while the drop-on-drop structure was determined using indexed

frames from ~5,000 3-5 μm crystals at room temperature, requiring measurements on ~50,000 drops containing ~10^6-10^7 crystals.

Movie descriptions:

Movie S1 Plunging of a MicroCrystal Mount #1 through a ligand solution film containing 5% w/v PEG 4000 and 10% v/v PEG 400, held in a 3 mm ID loop attached to a deposition stage as shown in **Figure 2**. The movie was recorded at 5000 frames per second. A substantial part of the side-to-side wobble is due to a small bend in the lead screw as purchased.

Movie S2 Plunging of a MicroCrystal Mount #1 through a ligand solution film containing 5% w/v PEG 4000 and 10% v/v PEG 400, held in a 2 mm ID loop attached to a deposition stage as shown in **Figure S8**. The movie was recorded at 4000 frames per second.

References

Adams, P. D., Afonine, P. V., Bunkóczi, G., Chen, V. B., Davis, I. W., Echols, N., Headd, J. J., Hung, L. W., Kapral, G. J., Grosse-Kunstleve, R. W., McCoy, A. J., Moriarty, N. W., Oeffner, R., Read, R. J., Richardson, D. C., Richardson, J. S., Terwilliger, T. C. & Zwart, P. H. (2010). *Acta Cryst. D* **66**, 213–221.

Amann, S. J., Keihsler, D., Bodrug, T., Brown, N. G. & Haselbach, D. (2023). *Structure* **31**, 4–19.

Berriman, J. & Unwin, N. (1994). *Ultramicroscopy* **56**, 241–252.

Bhattacharjee, B., Rahman, M. M., Hibbs, R. E. & Stowell, M. H. B. (2023). *Front. Mol. Biosci.* **10**, 1129225.

Bhattacharjee, S., Feng, X., Maji, S., Dadhwal, P., Zhang, Z., Brown, Z. P. & Frank, J. (2024). *Cell* **187**, 782-796.e23.

Butryn, A., Simon, P. S., Aller, P., Hinchliffe, P., Massad, R. N., Leen, G., Tooke, C. L., Bogacz, I., Kim, I.-S., Bhowmick, A., Brewster, A. S., Devenish, N. E., Brem, J., Kamps, J. J. A. G., Lang, P. A., Rabe, P., Axford, D., Beale, J. H., Davy, B., Ebrahim, A., Orlans, J., Storm, S. L. S., Zhou, T., Owada, S., Tanaka, R., Tono, K., Evans, G., Owen, R. L., Houle, F. A., Sauter, N. K., Schofield, C. J., Spencer, J., Yachandra, V. K., Yano, J., Kern, J. F. & Orville, A. M. (2021). *Nat. Comm.* **12**, 4461.

Calvey, G. D., Katz, A. M. & Pollack, L. (2019). *Analytical Chemistry* **91**, 7139–7144.

Chen, B., Kaledhonkar, S., Sun, M., Shen, B., Lu, Z., Barnard, D., Lu, T. M., Gonzalez, R. & Frank, J. (2015). *Structure* **23**, 1097–1105.

Clinger, J. A., Moreau, D. W., McLeod, M. J., Holyoak, T. & Thorne, R. E. (2021). *IUCrJ* **8**, 784–792.

Costello, M. J. (2006). *Ultrastruct Pathol* **30**, 361–371.

Cvetkovic, A., Picioreanu, C., Straathof, A. J. J., Krishna, R. & Van Der Wielen, L. A. M. (2005). *JACS* **127**, 875–879.

Dandey, V. P., Budell, W. C., Wei, H., Bobe, D., Maruthi, K., Kopylov, M., Eng, E. T., Kahn, P. A., Hinshaw, J. E., Kundu, N., Nimigean, C. M., Fan, C., Sukomon, N., Darst, S. A., Saecker, R. M., Chen, J., Malone, B., Potter, C. S. & Carragher, B. (2020). *Nature Methods* **17**, 897–900.

Deshchenya, V. I., Kondratyuk, N. D., Lankin, A. V. & Norman, G. E. (2022). *Journal of Molecular Liquids* **367**, 120456.

Ding, X., Rasmussen, B. F., Petsko, G. A. & Ringe, D. (2006). *Bioorganic Chemistry* **34**, 410–423.

Emsley, P., Lohkamp, B., Scott, W. G. & Cowtan, K. (2010). *Acta Cryst. D* **66**, 486–501.

Geremia, S., Campagnolo, M., Demitri, N. & Johnson, L. N. (2006). *Structure* **14**, 393–400.

Hajdu, J., Neutze, R., Sjögren, T., Edman, K., Szöke, A., Wilmouth, R. C. & Wilmot, C. M. (2000). *Nat. Struct. Bio.* **7**.

Kaledhonkar, S., Fu, Z., Caban, K., Li, W., Chen, B., Sun, M., Gonzalez, R. L. & Frank, J. (2019). *Nature* **570**, 400–404.

Klebl, D. P., White, H. D., Sobott, F. & Muench, S. P. (2021). *Acta Cryst. D* **77**, 1233–1240.

Kontziampasis, D., Klebl, D. P., Iadanza, M. G., Scarff, C. A., Kopf, F., Sobott, F., Monteiro, D. C. F., Trebbin, M., Muench, S. P. & White, H. D. (2019). *IUCrJ* **6**, 1024–1031.

Malla, T. N., Zielinski, K., Aldama, L., Bajt, S., Feliz, D., Hayes, B., Hunter, M., Kupitz, C., Lisova, S., Knoska, J., Martin-Garcia, J. M., Mariani, V., Pandey, S., Poudyal, I., Sierra, R. G., Tolstikova, A., Yefanov, O., Yoon, C. H., Ourmazd, A., Fromme, P., Schwander, P., Barty, A., Chapman, H. N., Stojkovic, E. A., Batyuk, A., Boutet, S., Phillips, G. N., Pollack, L. & Schmidt, M. (2023). *Nat Commun* **14**, 5507.

Martin-Garcia, J. M., Conrad, C. E., Nelson, G., Stander, N., Zatsepin, N. A., Zook, J., Zhu, L., Geiger, J., Chun, E., Kissick, D., Hilgart, M. C., Ogata, C., Ishchenko, A., Nagaratnam, N., Roy-Chowdhury, S., Coe, J., Subramanian, G., Schaffer, A., James, D., Ketwala, G., Venugopalan, N., Xu, S., Corcoran, S., Ferguson, D., Weierstall, U., Spence, J. C. H., Cherezov, V., Fromme, P., Fischetti, R. F. & Liu, W. (2017). *IUCrJ* **4**, 1–16.

Mehrabi, P., Muller-Werkmeister, H. M., Leimkohl, J. P., Schikora, H., Ninkovic, J., Krivokuca, S., Andricek, L., Epp, S. W., Sherrell, D., Owen, R. L., Pearson, A. R., Tellkamp, F., Schulz, E. C. & Dwayne Miller, R. J. (2020). *J .Synch. Rad.* **27**, 360–370.

Mehrabi, P., Schulz, E. C., Agthe, M., Horrell, S., Bourenkov, G., Von Stetten, D., Leimkohl, J.-P., Schikora, H., Schneider, T. R., Pearson, A. R., Tellkamp, F. & Miller, R. J. D. (2019). *Nat Methods* **16**, 979–982.

Mehrabi, P., Sung, S., Von Stetten, D., Prester, A., Hatton, C. E., Kleine-Döpke, S., Berkes, A., Gore, G., Leimkohl, J.-P., Schikora, H., Kollewe, M., Rohde, H., Wilmanns, M., Tellkamp, F. & Schulz, E. C. (2023). *Nat Commun* **14**, 2365.

Moffat, K. & Henderson, R. (1995). *Curr. Opin. Struct. Biol.* **5**, 656–663.

Moreau, D. W., Atakisi, H. & Thorne, R. E. (2019). *IUCrJ* **6**, 346–356.

Pandey, S., Calvey, G., Katz, A. M., Malla, T. N., Koua, F. H. M., Martin-Garcia, J. M., Poudyal, I., Yang, J.-H., Vakili, M., Yefanov, O., Zielinski, K. A., Bajt, S., Awel, S., Doerner, K., Frank, M., Gelisio, L., Jernigan, R., Kirkwood, H., Kloos, M., Koliyadu, J., Mariani, V., Miller, M. D., Mills, G., Nelson, G., Olmos, J. L., Sadri, A., Sato, T., Tolstikova, A., Xu, W., Ourmazd, A., Spence, J. C. H., Schwander, P., Barty, A., Chapman, H. N., Fromme, P., Mancuso, A. P., Phillips, G. N., Bean, R., Pollack, L. & Schmidt, M. (2021). *IUCrJ* **8**, 878–895.

Papasergi-Scott, M. M., Pérez-Hernández, G., Batebi, H., Gao, Y., Eskici, G., Seven, A. B., Panova, O., Hilger, D., Casiraghi, M., He, F., Maul, L., Gmeiner, P., Kobilka, B. K., Hildebrand, P. W. & Skiniotis, G. (2024). *Nature* **629**, 1182–1191.

Rampp, M., Buttersack, C. & Lüdemann, H.-D. (2000). *Carbohydrate Research* **328**, 561–572.

Schlichting, I. & Chu, K. (2000). *Curr. Opin. Struct. Biol.* **10**, 744–752.

Schmidt, M. (2013). *Advances in Condensed Matter Physics* **2013**, https://doi.org/10.1155/2013/167276.

Stoddard, B. L. (2001). *Methods* **24**, 125–138.

Tomadakis, M. M. & Sotirchos, S. V. (1993). *The Journal of Chemical Physics* **98**, 616–626.

Torino, S., Dhurandhar, M., Stroobants, A., Claessens, R. & Efremov, R. G. (2023). *Nat Methods* **20**, 1400–1408.

Vagin, A. & Teplyakov, A. (2010). *Acta Cryst. D* **66**, 22–25.

Walker, M., Trinick, J., White, H., Brenner, B., Kawai, M., White, H., Thomas, D., Pollack, G., Cooke, R. & Rayment, I. (1995). *Biophysical Journal* **68**, 87–91.

Winter, G., Waterman, D. G., Parkhurst, J. M., Brewster, A. S., Gildea, R. J., Gerstel, M., Fuentes-Montero, L., Vollmar, M., Michels-Clark, T., Young, I. D., Sauter, N. K. & Evans, G. (2018). *Acta Cryst. D* **74**, 85–97.
